# Development of a recombinase polymerase based isothermal amplification combined with lateral flow assay (HLB-RPA-LFA) for rapid detection of "*Candidatus* Liberibacter asiaticus"

**DOI:** 10.1371/journal.pone.0208530

**Published:** 2018-12-12

**Authors:** Dilip Kumar Ghosh, Sunil B. Kokane, Amol D. Kokane, Ashish J. Warghane, Manali R. Motghare, Sumit Bhose, Ashwani Kumar Sharma, M. Krishna Reddy

**Affiliations:** 1 Plant Virology Laboratory, ICAR-Central Citrus Research Institute, Nagpur, Maharashtra, India; 2 Department of Biotechnology, Indian Institute of Technology Roorkee, Roorkee, Uttarakhand, India; 3 Plant Virology Laboratory, ICAR-Indian Institute of Horticulture, Bengaluru, Karnataka, India; Instituto de Biologia Molecular y Celular de Plantas, SPAIN

## Abstract

Huanglongbing (HLB) or citrus greening is highly destructive disease that is affecting the citrus industry worldwide and it has killed millions of citrus plants globally. HLB is caused by the phloem limited, Gram negative, non-culturable, alpha-proteobacterium, ‘*Candidatus* Liberibacter asiaticus’. Currently, polymerase chain reaction (PCR) and real time PCR have been the gold standard techniques used for detection of ‘*Ca*. L. asiaticus’. These diagnostic methods are expensive, require well equipped laboratories, not user-friendly and not suitable for on-site detection of the pathogen. In this study, a sensitive, reliable, quick and low cost recombinase polymerase based isothermal amplification combined with lateral flow assay (HLB-RPA-LFA) technique has been developed as a diagnostic tool for detection of ‘*Ca*. L. asiaticus’. The assay was standardized by designing the specific primer pair and probe based on the conserved 16S rRNA gene of ‘*Ca*. L. asiaticus’. The assay was optimized for temperature and reaction time by using purified DNA and crude plant extracts and the best HLB-RPA-LFA was achieved at the isothermal temperature of 38°C for 20 to 30 min. The efficacy and sensitivity of the assay was carried out by using field grown, HLB-infected, HLB-doubtful and healthy citrus cultivars including mandarin, sweet orange cv. mosambi, and acid lime. The HLB-RPA-LFA did not show cross-reactivity with other citrus pathogens and is simple, cost-effective, rapid, user-friendly and sensitive. Thus, the HLB-RPA-LFA method has great potential to provide an improved diagnostic tool for detection of ‘*Ca*. L. asiaticus’ for the farmers, nurserymen, disease surveyors, mobile plant pathology laboratories, bud-wood certification and quarantine programs.

## Introduction

*‘Candidatus* Liberibacter spp.’ causes the economically devastating disease of citrus known as Huanglongbing (HLB) or citrus greening disease [1, 2]. The disease significantly damages the citrus industry worldwide by reducing the life span of citrus trees, their productivity and the quality of citrus fruits, with an estimated yield loss up to 30–100% [3, 4]). The causal pathogen is a non-culturable, Gram-negative, α-proteo bacteria limited to the phloem tissues of infected plants [1]. On the basis of 16S rRNA gene sequences, environmental conditions and temperature susceptibility, the causal pathogens have been shown to belong to three Liberibacter spp. namely ‘*Candidatus* Liberibacterasiaticus’ (*Ca*. L. asiaticus), ‘*Candidatus* Liberibacterafricanus’ (*Ca*. L. africanus) and ‘*Candidatus* Liberibacteramericanus’ (*Ca*. L. americanus) [1, 5]. Among these three species, ‘*Ca*. L. asiaticus’ is principally responsible for the devastating effects of low productivity and mortality of citrus plants in India [6, 7]. ‘*Ca*. L. asiaticus’ is a graft transmissible pathogen that is readily transmitted by grafting and budding at nurseries [8]. The pathogen is vectored by the citrus psyllid (*Diphorinacitri*) and transmitted by grafting in the nursery[9].

Symptom expression of HLB depends on the type of host, pathogen strain, rootstock-scion combination, age of the citrus tree, time of infection and environmental conditions [8]. HLB-affected trees show symptoms including open growth, stunting, twig dieback, sparse yellow foliage and severe fruit drop. The most distinctive and important diagnostic symptom of HLB is the existence of the ‘blotchy mottle’ symptom on leaves [1]. Fruits on HLB-affected citrus trees are smaller, poorly developed, lopsided and drop easily. On the fruits, the orange colour starts first at the penduncular (upper) end, at a time when the stylar end is still green, in contrast to the normal colour development. Hence, the disease is known as citrus greening. Small fruits with aborted seeds, low juice, bitter taste, low in soluble solids and high in acid content are the distinctive symptoms of HLB in fruits [8]. In severe case of HLB infection, ‘green island’ symptoms have been regularly observed on leaves of Mosambi (*Citrus sinensis*) plants [10]. Currently, no natural resistance in commercial cultivars or chemical control measures are available against ‘*Ca*. L. asiaticus’. Hence, rapid and user-friendly diagnostic tools are required to prevent its spread and take preventive control measures of the pathogen.

Numerous diagnostics techniques have been implemented for the detection of ‘*Ca*. L. asiaticus’ including biological indexing [11], enzyme-linked immunosorbent assay (ELISA) [12; [13], polymerase chain reaction (PCR) [14], real time-polymerase chain reaction (RT-PCR) [15, 16, 17, 18, 19], DNA-labeled hybridization probes [20] and loop mediated isothermal amplification (LAMP) [21, 22]. All the above mentioned techniques excluding LAMP require highly sophisticated instruments, trained personnel, costly molecular reagents and are time consuming. Therefore, there is an urgent need for the development of a technique that is rapid, user-friendly, sensitive and less costly.

The recombinase polymerase amplification (RPA) is an isothermal nucleic acid amplification technique. RPA reactions are carried out by using three enzymes, a single strand binding protein, a recombinase and a strand displacing DNA polymerase. Each enzyme performsa unique role in the reaction and operates at isothermal ranges between 25°Cand 40°C. Recombinase scans the primers and forms a nucleoprotein filament, which will bind to the complementary sequence on the template. The single strand binding protein then stabilizes the formed nucleoprotein filament in its corresponding position and the strand displacing DNA polymerase makes the extension of each strand, generating many copies of the template [23, 24, 25]. Recently, RPA has been combined with lateral flow assays (LFA) for rapid and visual detection of results. The LFA-based detection system requires an extra probe having a FAM residue at the 5′ end and reverse primers labeled with biotin on the 5′ end. Subsequently, the amplicons amplified by using these primers and probe are detected in the ‘sandwich’ assay [26]. Such type of detection device is available on the market *viz*, Nucleic acid detector PCRD and MileniaGenLineHybriDetect strip (TwistDx Limited, Cambridge, U K).

**Fig 1:**
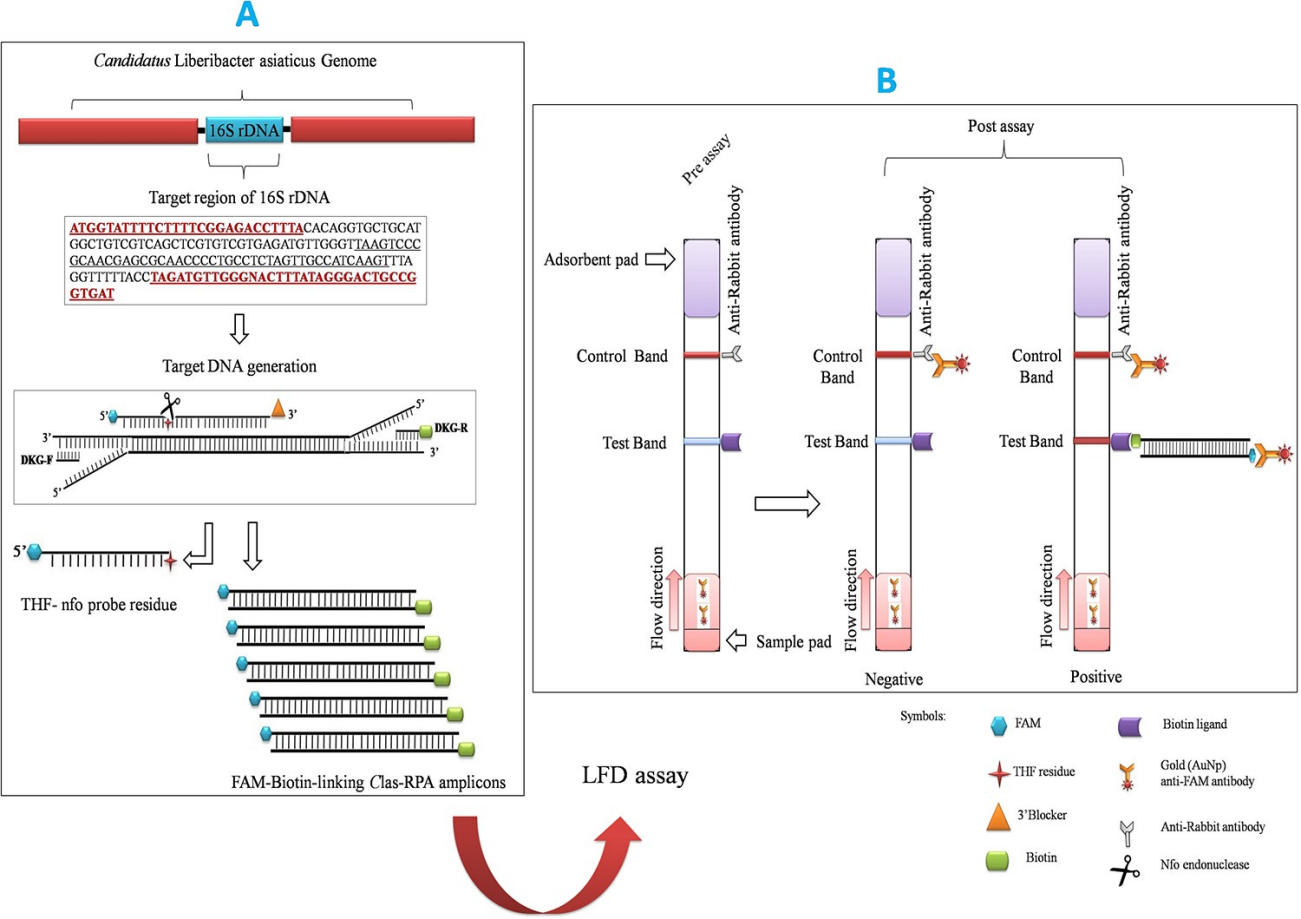
Schematic illustration of HLB-RPA-LFA principle for the detectionof ‘*Ca*. L. asiaticus’. (A) Amplification of FAM-biotin-linking ‘*Ca*. L. asiaticus’ 16S rRNA amplicons. The RPA driven primers (DKG-F/ Biotin labelled-DKG-R) first generate templates for the annealing of the nfo probe. In the resulting double strand context, nfo endonuclease present in the TwistAmpnfo kit recognizes the double-strand hybridization complex and cuts the THF residue. Polymerisation of ‘*Ca*. L. asiaticus’ above mentioned template using RPA enzymes. The DNA amplicons produced effectively co-join the two antigenic residues (FAM and biotin) in one DNA molecule. (B) Detection of FAM-Biotin linking ‘*Ca*. L. asiaticus’ RPA product by LFA using PCRD nucleic acid detector. The appearance of two red bands indicates the positive RPA assay for ‘*Ca*. L. asiaticus’ whereas appearance of a single red line indicates the negative results.

A number of RPA assays have been developed for rapid detection of plant pathogens such as *Yam mosaic virus* (YMV), *Yam mild mosaic virus* (YMMV) [27], *Phytophthora* spp. [28], *Citrus yellow mosaic virus* [29],*Tomato mottle virus*(ToMV), *Tomato yellow leaf curl virus* (TYLCV), *Bean golden yellow mosaic virus* (BGYMV) [30] and *Ca*. L. asiaticus[31]. The present study has focused on the development of a rapid and user friendly RPA combined with lateral flow assay-based diagnostic technique for detection of ‘*Ca*. L. asiaticus’ in citrus plants and its working principle is illustrated in figure (Fig 1).

## Materials and methods

### Plant source and sample processing

Six ‘*Ca*. L. asiaticus’ isolates (C1, C2, C3, C4, C5 and C6), originally collected from different geographical regions of India, have been maintained in Mosambi seedlings (*Citrus sinensis*) inside an insect proof screenhouse at ICAR-CCRI, Nagpur, Maharashtra, India and used as a positive control for RPA, PCR and real time PCR. The developed RPA technique was further validated by using eighty-eight HLB-doubtful samples collected from different geographical regions of India. Healthy citrus samples bought from an insect proof screenhouse were used as negative controls. For sampling, five to six leaves were collected from each plant into sterile polythene bags. The collected samples were washed with double distilled water, blotted dry and wiped using 70% ethanol to minimize any surface contamination.

### DNA extraction

Midribs and petiole from individual leaf samples were excised by scissor, cut into small pieces and ground in liquid nitrogen using mortar and pestle. Approximately 100 mg liquid nitrogen crushed sample were used for total DNA extraction by the DNeasy Plant mini kit (Qiagen, Germany). The 100 mg crushed samples were suspended with 400 μl buffer AP1 and 4 μl RNase A (100 mg/ml). The lysate was vortexed and incubated at 65°C for 10 min after which 130 μl buffer P3 was added. Samples were mixed properly, incubated on ice for 5 min and centrifuged at 20000×g for 5 min. The supernatant was added into a QIA shredder spin column placed in a 2 ml collection tube and centrifuged the lysate for 2 min at 20000×g. The flow-through was transferred into a new tube and mixed with 1.5 volumes of AW1 buffer. The 700 μl of the mixture was then transferred into a DNeasy mini spin column placed in a 2 ml collection tube, centrifuged for 1 min at ≥ 6000×g and after that flow-through was discarded. The 500 μl AW2 buffer was dispensed into a DNeasy mini spin column, centrifuged for 1min at ≥ 6000×g and flow-through was discarded. The above step was repeated with 2 min centrifugation at 20000×g. Finally, spin column was removed from the collection tube and transferred to a new 1.5 ml microcentrifuge tube and total DNA was eluted by adding AE buffer. The total genomic DNA from each sample was eluted in total 60 μl of elution buffer and quality and quantity was determined by using a NanoDrop 2000 Spectrophotometer (Thermo Scientific). The extracted DNA was stored at -20°C.

### Crude extraction

To develop an on-site diagnostic kit, the crude sap was prepared in AMP1 extraction buffer (ACC 00117/0020, Agdia, USA). Approximately 100 mg of midribs excised from fresh symptomatic leaf were ground in 1 ml of 1X AMP1 extraction buffer in a mortar and pestle until become fine paste, centrifuged at 9168 g (10000 rpm) for 2 min. The supernatant was taken out from particulate matter and used as a template for RPA reaction. Alternatively, the ground mixture was strained through two to three layers of muslin cloth and 1–2 μl of filtrate used as template.

### Primer and nfo-probe design for RPA-LFA

The primers and probe used in the HLB-RPA-LFA was designed using TwistAmpnfo assay design manual guidelines (www.twistdx.co.uk) to amplify a 170bp region specific to the 16S rRNA gene of ‘*Ca*. L. asiaticus’. To design the primers and probe, the most reliable and conserve region from 16S rRNA were selected by performing nucleotide BLAST of Indian Poona strain (GenBank L22532) with other strains available in NCBI database *viz*, strain A4, gxpsy, JXGC, AHCA-1, and Ishi-1 etc(www.ncbi.nlm.nih.gov) [29, 32]. For RPA primer design, the standard parameters *viz*. amplicon size, primer length, GC contents, pyrimidine at 5’ end and a G/C clamp were taken into consideration. The *in silico* specificity of DKG-F/ DKG-R primer was investigated using primer-BLAST software (www.ncbi.nlm.nih.gov)[33]. The *in silico* PCR was also performed against different *Candidatus* species (http://www.in-silico.com) (S1 Table). The PCR was simulated against up-to-date sequenced prokaryotic genomes. This analysis allows a maximum of 2 mismatches between primers and template, so the stringency should be considered high [34].

### Conventional PCR

To confirm the presence of ‘*Ca*. L. asiaticus’, PCR amplification was carried out by using 16S rDNA-based primer sets OI1/OI2c (Table 1), in 25 μl reaction volume. Each reaction contained 1 μl total DNA, 1X PCR buffer, 1.5 mM MgCl_2_, 0.2 mM of dNTP each, 0.2 μM of each primer, 1.25U of GoTaq DNA polymerase (Promega) and nuclease-free water. PCR amplification was carried out in My Cycler (Bio-Rad, Hercules, CA, USA) with one cycle of 3 min at 94°C followed by 35 cycles of 30 s at 94°C, 1 min at 58°C, 1.30 min at 72°C, and final extension at 72°C for 10 min. The PCR amplified products were checked by 1% agarose gel electrophoresis using 1 kb marker (BioLabs). Another primer set, A2/J5specific to rplA-rpl J region of ‘*Ca*. L. asiaticus’ also used to validate the RPA technique (Table 1). It corresponds to the conserved β operon’s subunits of 50S ribosomal protein gene [35, 36]. PCR amplification using A2/J5 primer set was carried out in 25μl reaction volume with one cycle of 3 min at 94°C followed by 35 cycles of 30 s at 94°C, 45 s at 53°C, 1 min at 72°C, and final extension at 72°C for 10 min. The PCR amplified products were visualized by 1% agarose gel electrophoresis using 100bp marker (BioLabs).

**Table 1:** Conventional PCR primers used in present study.

Sr. no	Primer Code	Sequence (5′- 3′)	Product Size	Reference
**1**	OI1	GCGCGTATGCAATACGAGCGGCA	1160bp	[14]
**2**	OI2c	GCCTCGCGACTTCGCAACCCAT		
**3**	A2	TATAAAGGTTGACCTTTCGAGTTT	703bp	[36]
**4**	J5	ACAAAAGCAGAAATAGCACGAACAA		

### TaqMan-qPCR

TaqMan-qPCR was performed to compare and confirm the detection limit of the RPA-lateral flow assay. To verify whether the samples (C1 to C6) were positive for ‘*Ca*. L. asiaticus’, the extracted DNA was also analyzed by TaqMan-real time PCR with the HLBas-F/R-HLBp primers-probe combination[37, 38, 39]. The evaluated sensitivity of the RPA-LFA towards the ‘*Ca*. L. asiaticus’ were cross verified with real time PCR assay using TaqMan chemistry (S1 File). The same 1:10 serially diluted genomic DNA (10 ng, 1 ng, 0.1 ng, 0.01 ng, 0.001 ng, 0.0001 ng, 0.00001 ng) from *C*Las infected Mosambi plants were used for cross verification with TaqMan-real time PCR. All reactions were performed in triplicate along with non-template controls.

### Conventional PCR with RPA primers

To validate the specificity of RPA primers, conventional PCR was run with newly- designed RPA primers (Table 2). The total reaction was carried out in 25 μl volumes, containing 1X PCR buffer, 1.5mM MgCl2, 0.2 mM of dNTP each, 0.2μM of each primer, 1.25U of GoTaq DNA polymerase (Promega) and nuclease-free water. PCR amplification was carried out as follows: one cycle of 3 min at 94°C followed by 35 cycles that comprised, 30 s at 94°C, 40s at 58°C, 45 s at 72°C, and final extension at 72°C for 10 min. The PCR amplified products were checked by 1.5% agarose gel electrophoresis with 100 bp marker.

**Table 2:** RPA primers and probe used in present study.

Sr. no	Primer / Probe Code	Sequence (5′- 3′)	Product Size
**1**	DKG-F	GATGGTATTTTCTTTTCGGAGACCTTTACA	170bp
**2**	DKG-R	[Btn] ATCACCGGCAGTCCCTATAAAGTACCCAACATCTA	
**3**	HLB-Probe	[6FAM]TAAGTCCCGCAACGAGCGCAACCCCTGCCT(H)TAGTTGCCATCAAGT-Spacer C3	

(Btn) = Biotin, [6FAM] = FAM flurophore, (H) = Tetrahydrofuran (THF) residue

### Recombinase polymerase amplification

Recombinase polymerase amplification reaction was carried out using the designed primers DKG-F/DKG-R. The reaction was performed using the manufacturer’s protocol with slight modification as outlined in the Twist Amp basic kit (TwistDx, Cambridge, UK). The reaction was performed in 50 μl reaction volumes containing 2.4 μl of each primer DKG-F/DKG-R-Btn(0.48μM), 29.5 μl rehydration buffer, 1 μl DNA template and 12.2 μl nuclease free water. The contents were vortexed briefly for few seconds and the reaction mixture was added to the freeze-dried reaction provided and gently mixed by pipetting. Next, 2.5 μl of MgAc(14mM)(supplied) was added and mixed well to start the reaction. The reaction tube was incubated at 38°C for 25 min in a Dry bath(Labtech, Woodbridge, USA). After 4 min of incubation, the reaction tube was removed, gently vortexed, centrifuged for a few seconds and the incubation continued for a specified time. After the completion of the reaction, the RPA products were heat inactivated at 65°C for 10 min and prior to loading on 1.5% agarose gel to visualize it was mixed with 5% SDS. To obtain better band visibility on the agarose gel, amplification products were purified by initially diluting1:10 in water followed by extraction with a mixture of chloroform/isoamyl alcohol (24:1). The RPA amplified products were checked by 1.5% agarose gels with 100 bp marker.

### RPA-LFA

The recombinase polymerase reaction-lateral flow assay was carried out by using TwistAmpnfo kit (Cambridge, UK) and PCRD nucleic acid detector (Abingdon Health, York, UK). The reaction was carried out in 50 μl reaction volumes containing 2.1 μl of each primer DKG-F/DKG-R-Btn (0.42μM), 0.6 μl HLB- probe (0.12μM), 29.5 rehydration buffer, 1μlDNA template and 12.2 μl nuclease free water. The reaction tube was vortexed gently, centrifuged, and the reaction mixture was added to the provided freeze-dried reaction, mixed by pipetting and2.5 μl of MgAc(14mM) was added to start the reaction. The reaction tube was incubated at 38°C for 25 min (Dry bath, Labtech, Woodbridge, USA). After 4 min of incubation, the reaction tube was removed, vortexed, and incubation continued for a specified time. For LFA analysis, the reaction product was diluted (0.75–2:75) with the buffer (PCRD extraction buffer) and the entire 75μl of diluted reaction loaded onto the sample pad of the PCRD nucleic acid detector (Abingdon Health, York, UK) and the results were observed after 5 min.

The optimization of HLB-RPA-LFA was carried out using DNA as well as crude extract of symptomatic leaf midribs. The supernatant or filtrate was directly used as a template. To determine the optimum amplification temperature for the HLB-RPA-LFA, the assay was performed at temperatures of 20°C, 25°C, 30°C, 35°C, 37°C, 38°C, 40°C and 42°C and the optimum reaction time was determined by incubating the reaction mixtures for 0, 5, 10, 15, 20, 25, 30, 35 and 40 min after the addition of magnesium acetate. Validation of the HLB-RPA-LFA assay was performed using the AmplifyRP Acceler8 Las detection kit (Agdia, USA) as per manufacturer’s directions.

### Specificity and sensitivity of RPA-LFA

The specificity and sensitivity of the HLB-RPA-LFA towards the target pathogen were evaluated. The sensitivity of RPA assay was investigated using PCRD nucleic acid detector. The amplification efficiency ofDKG-F/ DKG-R-Btn primer was determined by use of 1:10 serially diluted genomic DNA (10 ng, 1 ng, 0.1 ng, 0.01 ng, 0.001 ng, 0.0001 ng, 0.00001 ng) from *C*Las infected Mosambi plants. The specificity and uniqueness were also evaluated using genomic DNA of *C*Las from HLB positive, HLB-doubtful samples and major virus and bacterial pathogen infecting citrusby conducting HLB-RPA-LFA and compared their specificity with other detection technique (Table 3). The sensitivity of RPA-LFA also compared with conventional PCR using primer set OI1/OI2c, A2/J5 andTaqMan-qPCR with primer pair (HLBas-F/R) and probe (HLBp).

## Results

### Primer specificity

*In silico* analysis was performed for specificity and cross reactivity of RPA-LFA primers using the genome of different *Candidatus* species and other citrus pathogens with primer-BLAST software. It was observed that primers were unable to find complementary regions when allowing primer specificity stringency, a total 2 mismatches to unintended targets against any citrus infecting pathogen except ‘*Ca*. L. asiaticus’. *In silico* PCR also performed against different *Candidatus* species using DKG-F/ DKG-R primer (S1 Table) and primers failed to amplify a target region of 170bp against sequenced genomic DNA of 70 non-target *Candidatus* species. However, 170 bp band was observed for ‘*Ca*. L. asiaticus’str. psy62 and ‘*Ca*. L. asiaticus’str. gxpsy. *In silico* analysis of the primers DKG-F/ DKG-R clearly ensured specificity of the RPA-LFA technique for ‘*Ca*. L. asiaticus’ detection.

**Fig 2:**
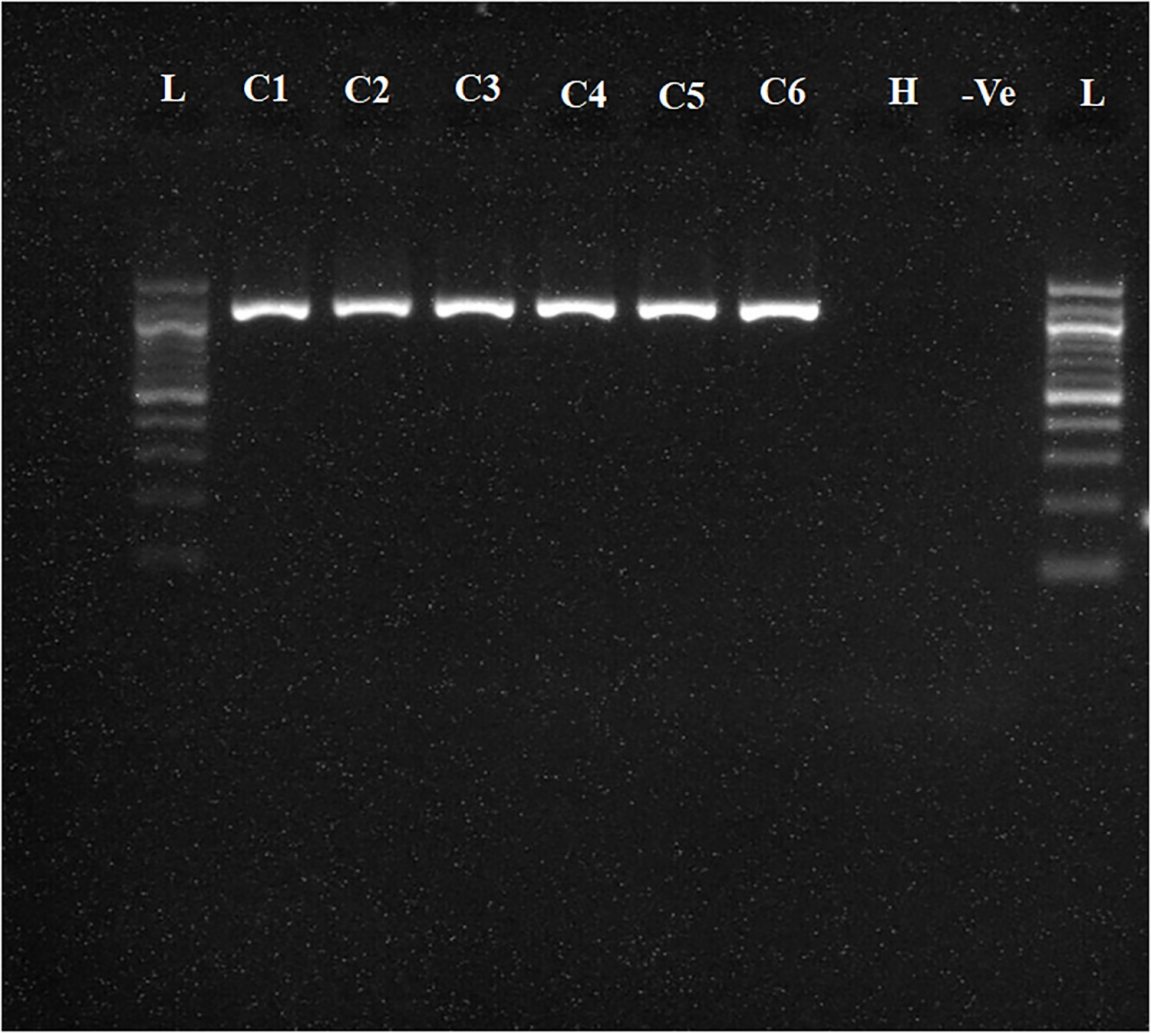
Agarose gel electrophoresis of PCR products. **Amplified products using OI1/OI2c ‘*Ca*. L. asiaticus’ specific primers were separated by 1% agarose gels**. Observed amplification product of 1160 bp product is shown.C1 to C6 represent the ‘*Ca*. L. asiaticus’ isolates maintained in the screenhouse. H: Healthy control; -Ve: negative control and L: 1 kb marker.

**Fig 3:**
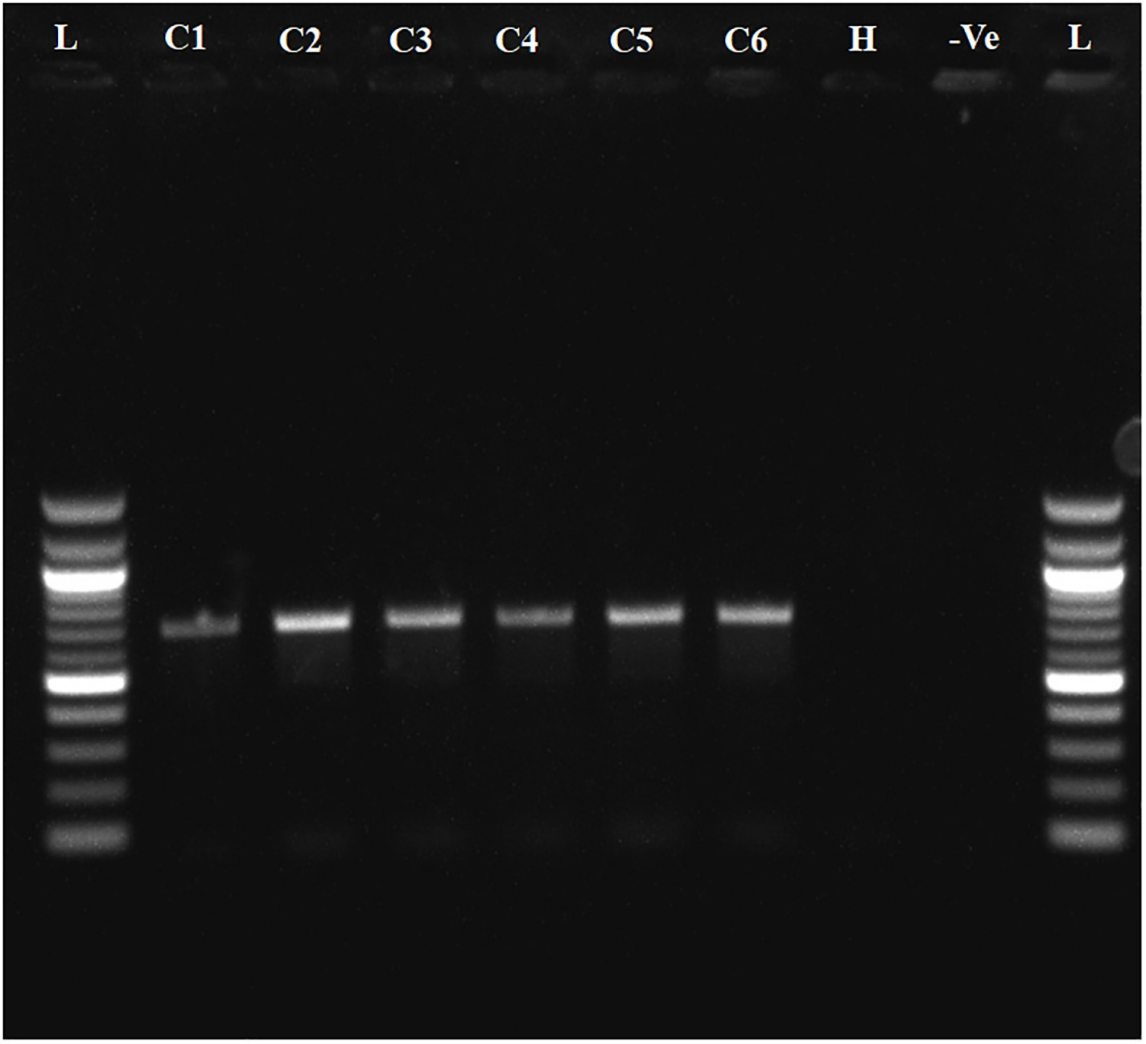
Agarose gel electrophoresis of PCR products. **Amplified products using A2/J5 ‘*Ca*. L. asiaticus’ specific primers were separated by 1% agarose gels**. Observed amplification product of 703bp product is shown. C1 to C6 represent the ‘*Ca*. L. asiaticus’ isolates maintained in the screenhouse. H: Healthy control; -Ve: negative control and L: 100bp marker.

**Fig 4:**
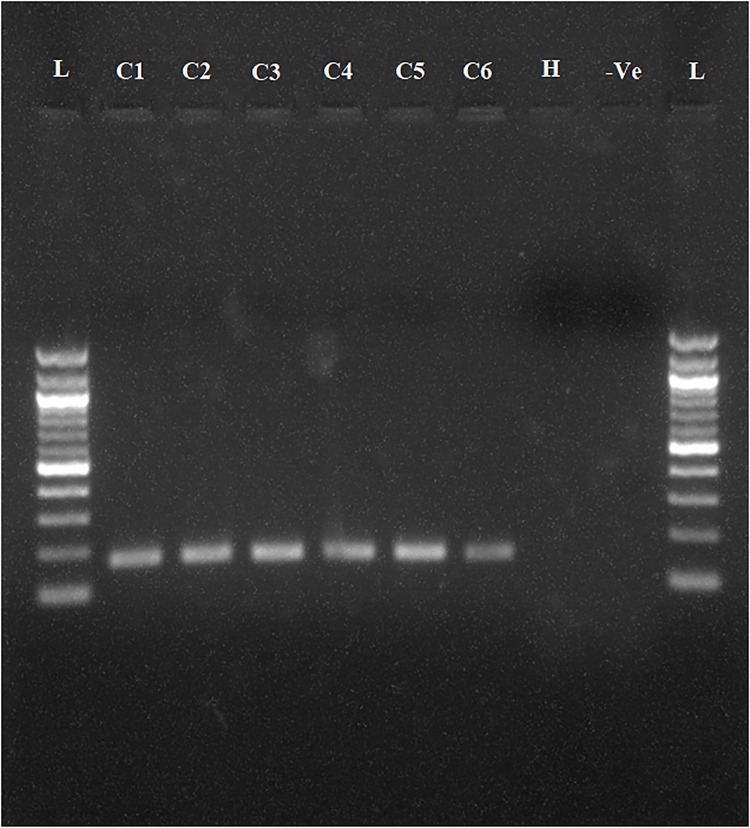
Agarose gel electrophoresis (1.5%) of PCR products amplified using DKG-F/DKG-R ‘*Ca*. L. asiaticus’ specific primers. Observed amplification of 170 bp product is shown.C1 to C6 represent the ‘*Ca*. L. asiaticus’ isolates maintained in the screenhouse. H: Healthy control, -Ve: negative control and L: 100 bp marker.

To determine whether the collected samples were positive for ‘*Ca*. L. asiaticus’, the extracted DNA was analyzed by conventional PCR with the 16S rDNA-based OI1/ OI2c primers and rplA-rplJ region based A2/J5 primers. An expected, a1160 bp amplicon for OI1/ OI2c primers and 703bp for A2/J5 primers was observed in samples C1 to C6 upon agarose gel electrophoresis and no band was recorded in the healthy sample (H) and reaction control(Figs 2 and 3). To check the specificity and efficacy of the RPA primers (DKG-F/DKG-R),conventional PCR was first undertaken with RPA primers. The expected amplification product of 170 bp was observed after electrophoresis in ‘*Ca*. L. asiaticus’ positive samples (C1 to C6) whereas no amplification was observed in the healthy samples (H) and reaction control (Fig 4). The RPA products amplified by RPA primers (DKG-F/DKG-R) were purified by gel elution and sequenced by Sanger dideoxy method (Eurofins genomics, Bengaluru, India) with quality score Q20. The chromatograms of both forward and reverse sequences were opened with bioedit (Bioedit Sequence Alignment Editor) for easy visualizing the sequence peak and reverse complement of the reverse sequence was made. The reliable sequence was selected by eliminating sequence having very low peak at both ends of the sequence. The forward sequence and reverse sequence were assembled into one complete contiguous sequence by eliminating repeated sequence and nucleotide BLAST was performed. The BLASTn result showed 99% query cover, 99% sequence identity and E-value (8e-79) with all strains *viz*. AHCA1 (CP029348), JXGC (CP019958), Ishi-1 (AP014595), gxpsy (CP004005) and the Sey8 isolate (MG212527). The above observation suggested that the RPA primers specifically amplified the 16S rRNA region of ‘*Ca*. L. asiaticus’.

### HLB-RPA

**Fig 5:**
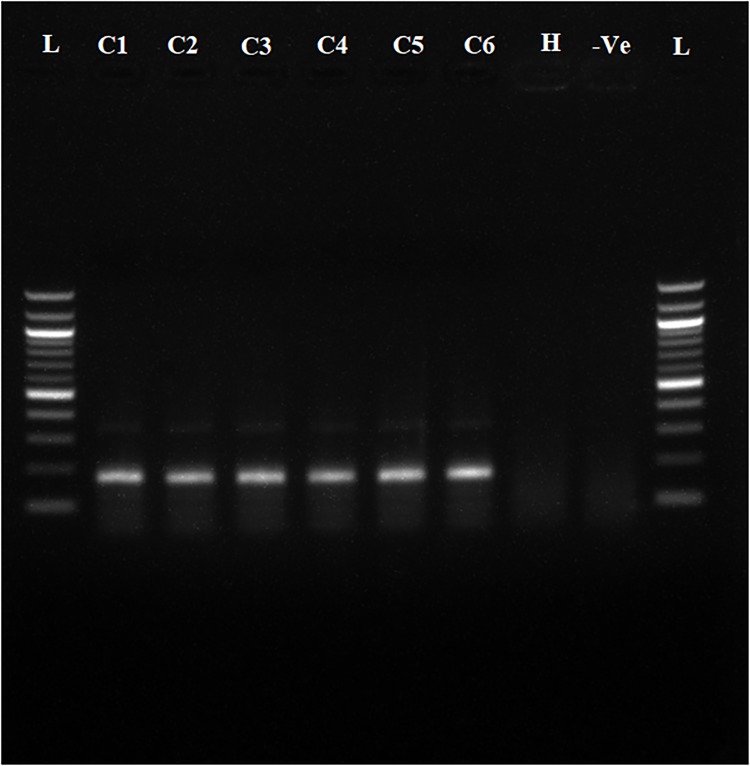
Agarose gel electrophoresis of recombinase polymerase amplification products amplified using DKG-F/DKG-R ‘*Ca*. L. asiaticus’ specific primers. Observed amplification of 170 bp product is shown.C1 to C6 represent the ‘*Ca*. L. asiaticus’ isolates maintained in the screenhouse, H: Healthy control, -Ve: negative control and L: 100bp marker.

Total DNA isolated (see above) from HLB positive and negative citrus plants was used for standardization of the HLB-RPA assay. The RPA assay gave best results at 38°Cwith an extension time of 20–30 min. Agarose gel electrophoresis of the RPA assay product showed the expected 170 bp band in the HLB positive samples (C1 to C6) whereas no band was observed in the healthy citrus plants (H) and the reaction control (Fig 5). In addition to an expected amplicon, a very faint additional ~350 bp bands was observed after electrophoresis in positive samples.

### HLB-RPA-LFA

**Fig 6:**
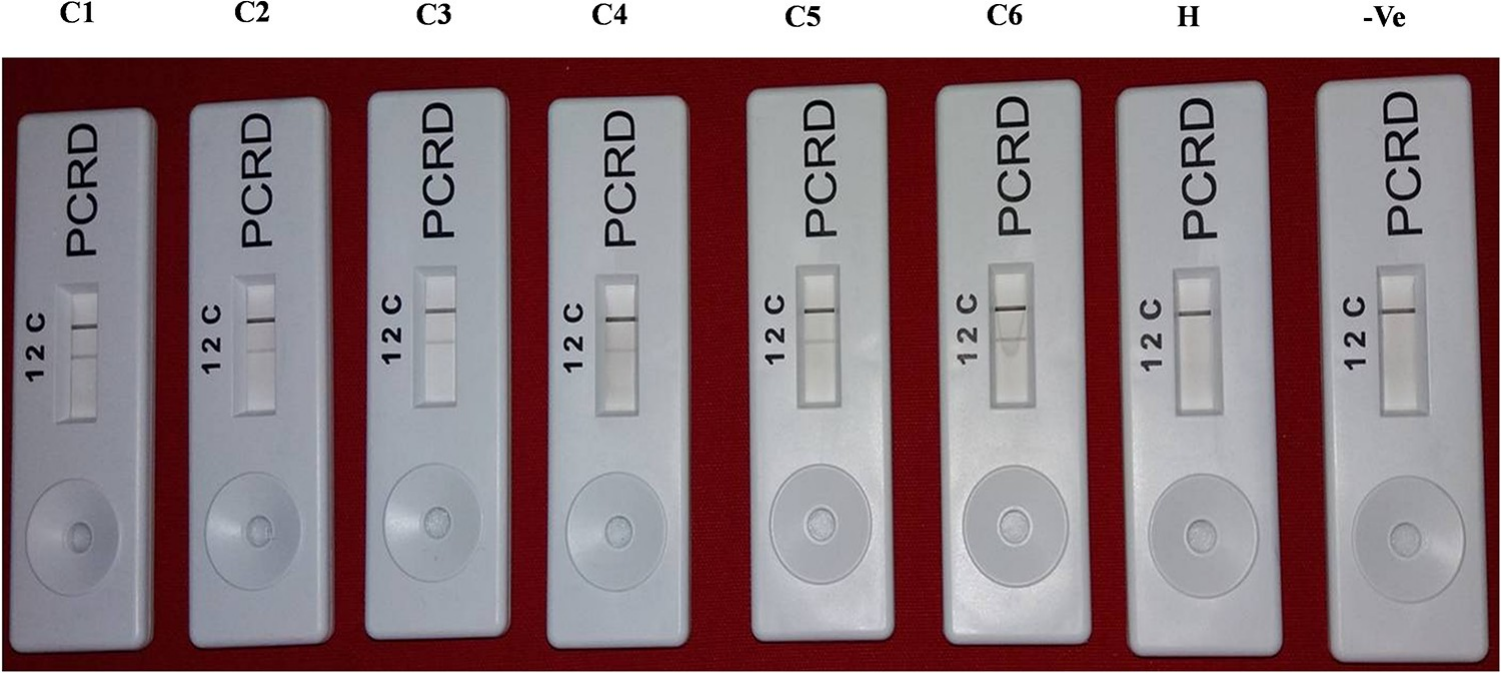
HLB-RPA-LFA; Huanglongbing-recombinase polymerase amplification-lateral flow assay with DNA as a template, showing three reaction lines: C is control; 2 is for detection of FAM/Biotin-labelled HLB amplicons. **(1 is not used in present assay)**. C1 to C6 represents the ‘*Ca*. L. asiaticus’ isolates maintained in the screenhouse, H: Healthy control and -Ve: negative control.

**Fig 7:**
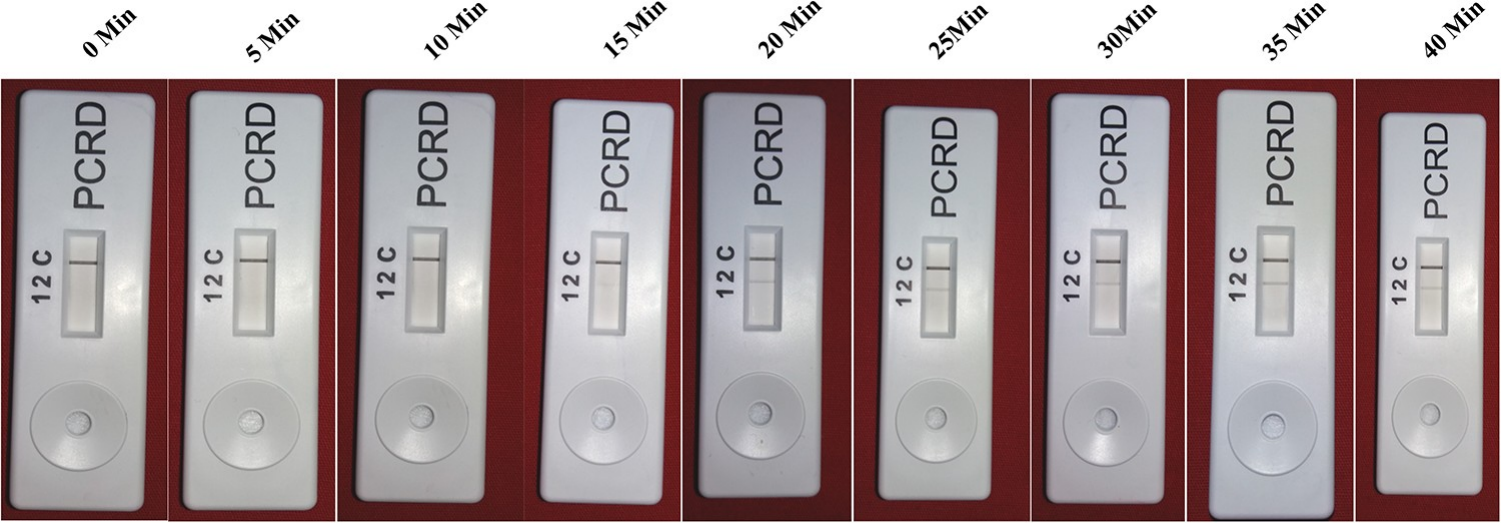
HLB-RPA-LFA, Determination of optimum reaction time. The best visibility of the test line was observed in the ranges20, 25, 30 and 35 min.

**Fig 8:**
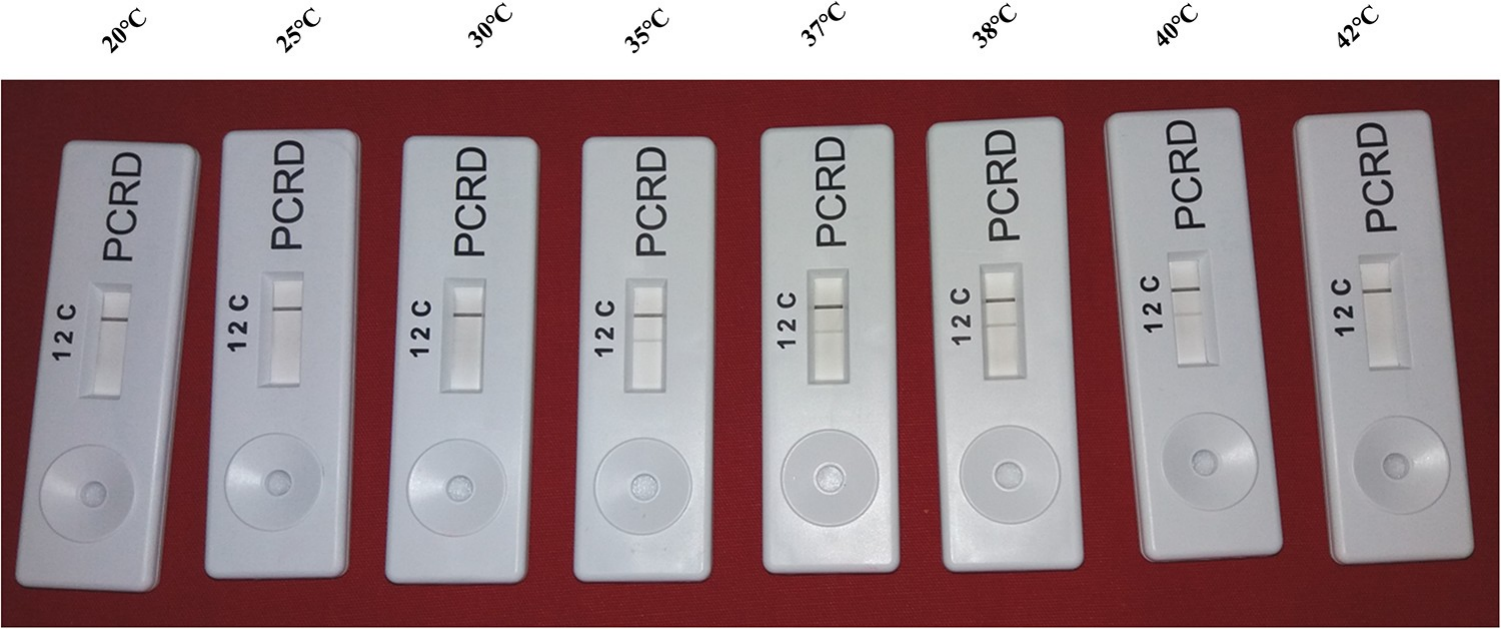
HLB-RPA-LFA, Determination of optimum reaction temperature. The best visibility of the test line was observed in the ranges 35°C, 37°C, 38°C and 40°C.

The screenhouse maintained‘*Ca*. L. asiaticus’ isolates (C1, C2, C3, C4, C5 and C6) and healthy (HLB negative) plants were used as a positive control and negative control respectively, during development of HLB-RPA-LFA. The optimization of RPA-LFA reaction was performed by two methods, first based on DNA as a template extracted by DNeasy Plant mini kit and in other the supernatant of crude sap was used directly as a template. In order to develop easy, rapid and visual detection of ‘*Ca*. L. asiaticus’, a PCRD nucleic acid detector was used to capture amplified products of RPA-LF Probe. After completion of the RPA, the diluted mixture was applied on the pad of the PCRD detector. Visualization of two bands (lines), a control line and the test line showed the presence of the pathogen in the citrus samples (Fig 6) while development of only the control line indicated absence of ‘*Ca*. L. asiaticus’ as observed with healthy and negative controls. It was observed that the bands started developing after 3 min and completed after 5 min in the test line as well as in the control line. It was observed that the intensity of the test line was less as compared to the control line, which may be due to the low titer of pathogen in test samples. In crude sample preparations (plant extract), the test band was less intense as compared to the DNA purified by the DNeasy Plant mini kit. This may be due to the presence of phenolic compounds in the citrus plant extract which may inhibit the RPA enzyme that inhibit the accessibility of RPA enzyme with the template DNA. Therefore, further validation of RPA technique was accomplished by using various numbers of HLB-doubtful samples collected from different geographical regions based on DNA as a template. The best optimal conditions for the visibility of the test line were at a reaction time 20 to 30 min and incubation temperature at 38°C (Figs 7 and 8). However, the faint test line was observed after incubation time of 15 min. All further reactions were performed by use of these optimal temperature and time parameters.

### Specificity and sensitivity of RPA-LFA

**Fig 9:**
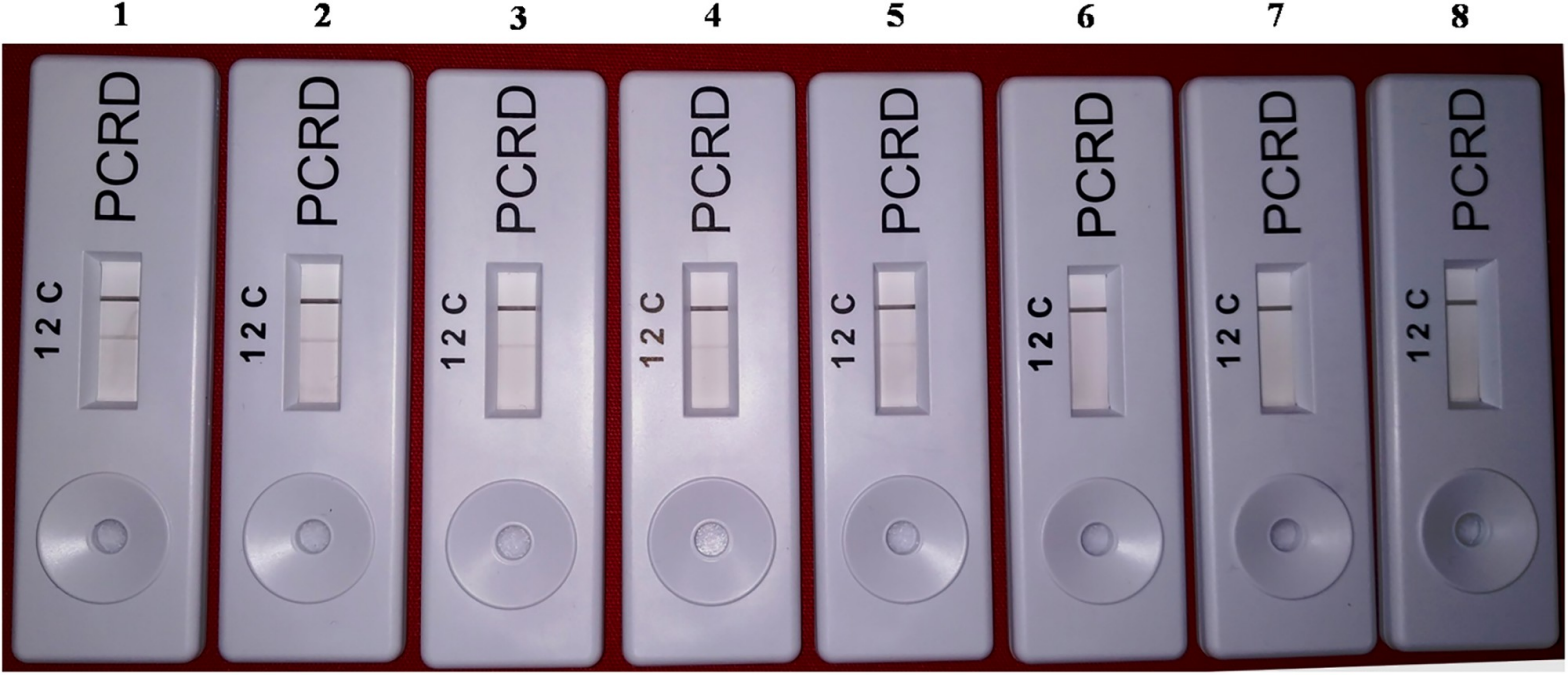
Detection limit of HLB-RPA-LFA. The amplified RPA products were detected on PCRD strip. The order of samples is lanes 1–7, amplified RPA products with concentrations 10 ng/μl, 1ng/μl, 0.1 ng/μl, 10 pg/μl, 1 pg/μl, 0.1 pg/μl, 0.01 pg/μl template DNA of HLB infected plant. Lane 8, NTC (non template control).

**Fig 10:**
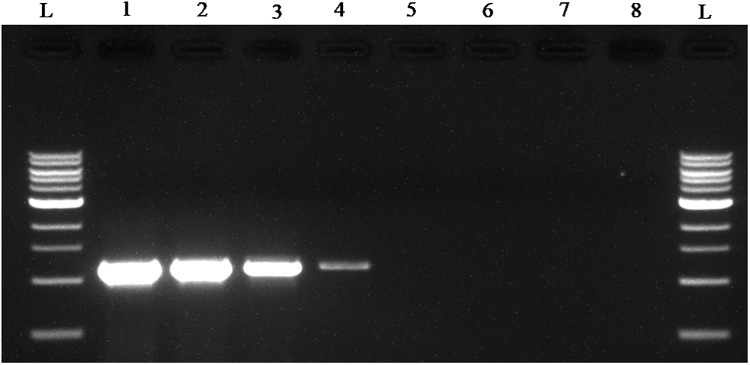
Detection limit of conventional PCR using OI1/OI2c ‘*Ca*. L. asiaticus’ specific primers. The amplified PCR products are resolved on 1% agarose gel. The order of samples is lane L, 1kb DNA marker; lanes 1–7, amplified PCR products with concentrations 10 ng/μl, 1 ng/μl, 0.1 ng/μl, 10 pg/μl, 1 pg/μl, 0.1 pg/μl, 0.01 pg/μl template DNA of HLB infected plant. Lane 8, NTC (non template control).

The standardized RPA-LFA consistently detected *C*Las from extracted DNA at concentrations ranging from 1pg to 1x 10^3^pg. The assay showed test band intensity approximately parallel with initial template concentration, high template concentration produce good intensity band as compare to low template concentration (Fig 9). The detection limit of RPA-LFA was ≤ 1 pg of total DNA from *C*Las infected plant. The limit of detection for conventional PCR in our analysis was recorded up to 10 pg (Fig 10). Whereas the TaqMan real-time PCR assay was able to detect DNA concentrations as low as 100–10 fg of *C*Las (S1 Fig). The standardized RPA-LFA using DKG-F/ DKG-R were highly specific for *C*Las. The results showed that primers failed to amplify genomic DNA of non-target bacterial species and did not generate any test band with genomic DNA from non-target pathogens.

### Validation of RPA assays using field samples

**Fig 11:**
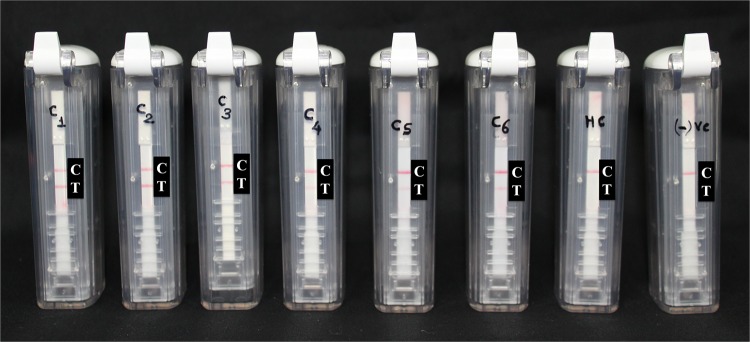
Validation of HLB-RPA-LFA assay by using AmplifyRP Acceler8 Las detection kit showing two reaction lines: C is control and T is the test line. C1 to C6 represents the ‘*Ca*. L. asiaticus’ isolates maintained in the screenhouse, H: Healthy control and -Ve: negative control.

**Fig 12:**
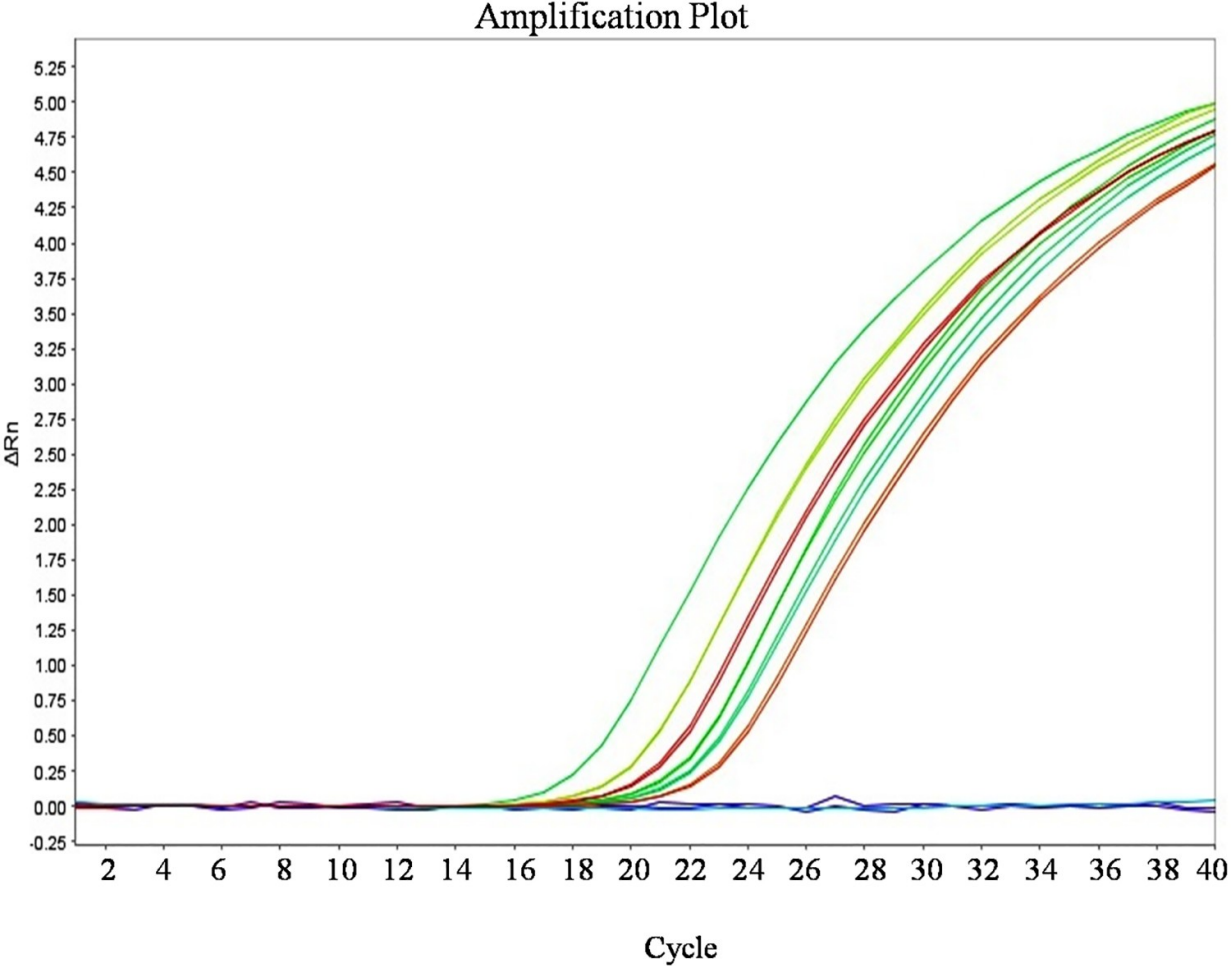
Validation of HLB-RPA-LFA assay by using TaqMan-qPCR with HLBas-F/R-HLBp primer probe pair. Amplification plot for sample C1 to C6 represent the ‘*Ca*. L. asiaticus’ isolates showing average Ct value, 23.29, 18.78, 22.23, 22.76, 21.50 and 20.62 respectively whereas undetermined Ct observed for non template control and H: Healthy control.

The validation of the developed HLB-RPA-LFA was carried out by using the AmplifyRP Acceler8 Las detection kit with the samples collected from the different locations throughout India (C1 to C6) and healthy controls. The developed HLB-RPA-LFA kit work truthfully and it was cross validated by using Amplify RP Acceler8 kit and TaqMan-qPCR with primer pair (HLBas-F/R) and probe (HLBp). Both techniques reflect the same results for all collected samples (Figs 11 and 12). The results of assay were further validated by amplification using conventional PCR.

To determine the efficacy of the developed HLB-RPA-LFA, eighty-eight samples, which was doubtful for HLB were collected from the different geographical regions of India and each sample was checked separately by HLB-RPA-LFA, HLB-RPA, PCR with RPA primers and conventional PCR for the presence of ‘*Ca*. L. asiaticus’ using DNA as a template. Out of these, thirty-eight samples were found positive with all four techniques (Table 3). Six healthy samples, one for each cultivar Nagpur mandarin (*Citrus reticulata*), Acid lime (*Citrus aurantifolia*), Sweet orange (*Citrus sinensis*), Pummelo (*Citrus grandis)*,Grapefruit (*Citrus paradisi*) and Assam Lemon (*Citrus limon*) were also checked and confirmed as a healthy plant (HLB- negative)(Table 3).

**Table 3:** Specificity of HLB-RPA-LFA, HLB-RPA, PCR with RPA primers and conventional PCR for the detection of ‘*Ca*. L. asiaticus’.

Sr. No	Sample code	Cultivar	Location	Target pathogen	Checked for *“Ca*. L. asiaticus”			
					HLB-RPA-LFA	HLB-RPA	PCR with RPA primers	Conventional PCR
**1**	An-3	Sweet orange (*Citrus sinensis*)	Allahabad, Utter Pradesh	*Ca*. L. asiaticus	**+**	**+**	**+**	**+**
**2**	An-4	Sweet orange (*Citrus sinensis*)	Allahabad, Utter Pradesh	*Ca*. L. asiaticus	**+**	**+**	**+**	**+**
**3**	An-5	Sweet orange (*Citrus sinensis*)	Allahabad, Utter Pradesh	*Ca*. L. asiaticus	**+**	**+**	**+**	**+**
**4**	TN-2	Acid lime (*Citrus aurantifolia*)	Perikulam, Tamil Nadu	*Ca*. L. asiaticus	**+**	**+**	**+**	**+**
**5**	TN-3	Acid lime (*Citrus aurantifolia)*	Perikulam, Tamil Nadu	*Ca*. L. asiaticus	**+**	**+**	**+**	**+**
**6**	TN-5	Acid lime (*Citrus aurantifolia*)	Perikulam,Tamil Nadu	*Ca*. L. asiaticus	**+**	**+**	**+**	**+**
**7**	AP-6	Sour orange (*Citrus aurantium*)	Tirupati, Andhra Pradesh	*Ca*. L. asiaticus	**+**	**+**	**+**	**+**
**8**	AP-7	Sour orange (*Citrus aurantium*)	Tirupati, Andhra Pradesh	*Ca*. L. asiaticus	**+**	**+**	**+**	**+**
**9**	AP-9	Madam vinous (*Citrus sinensis*)	Tirupati, Andhra Pradesh	*Ca*. L. asiaticus	**+**	**+**	**+**	**+**
**10**	CK-1	Sour orange (*Citrus aurantium*)	Chettali, Karnataka	*Ca*. L. asiaticus	**+**	**+**	**+**	**+**
**11**	CK-2	Grapefruit (*Citrus paradisi*)	Chettali, Karnataka	*Ca*. L. asiaticus	**+**	**+**	**+**	**+**
**12**	CK-5	Coorg mandarin (*Citrus reticulata*)	Chettali, Karnataka	*Ca*. L. asiaticus	**+**	**+**	**+**	**+**
**13**	Sm-W-5	Sikkim mandarin (*Citrus reticulata*)	West, Sikkim	*Ca*. L. asiaticus	**+**	**+**	**+**	**+**
**14**	Sm-W-8	Sikkim mandarin *(Citrus reticulata)*	West, Sikkim	*Ca*. L. asiaticus	**+**	**+**	**+**	**+**
**15**	Sm-S-7	Sikkim mandarin (*Citrus reticulata*)	South, Sikkim	*Ca*. L. asiaticus	**+**	**+**	**+**	**+**
**16**	Masl-1	Assam Lemon (*Citrus limon*)	Tuivamit, Mizoram	*Ca*. L. asiaticus	**+**	**+**	**+**	**+**
**17**	Mpml-2	Pummelo (*Citrus grandis)*	Tuivamit, Mizoram	*Ca*. L. asiaticus	**+**	**+**	**+**	**+**
**18**	Mmnd-1	Mandarin (*Citrus reticulata*)	Tuivamit, Mizoram	*Ca*. L. asiaticus	**+**	**+**	**+**	**+**
**19**	Km/Brg	Khasi mandarin (*Citrus reticulata*)	Tinsukia, Assam	*Ca*. L. asiaticus	**+**	**+**	**+**	**+**
**20**	So/crs	Sweet orange (*Citrus sinensis*)	Assam	*Ca*. L. asiaticus	**+**	**+**	**+**	**+**
**21**	Rl/crs	Rough lemon (*Citrus jumbhiri)*	Assam	*Ca*. L. asiaticus	**+**	**+**	**+**	**+**
**22**	NRC-24	Nagpur mandarin (*Citrus reticulata*)	Nagpur, Maharashtra	*Ca*. L. asiaticus	**+**	**+**	**+**	**+**
**23**	NRC-49	Nagpur mandarin (*Citrus reticulata*)	Nagpur, Maharashtra	*Ca*. L. asiaticus	**+**	**+**	**+**	**+**
**24**	NRC-66	Nagpur mandarin (*Citrus reticulata*)	Nagpur, Maharashtra	*Ca*. L. asiaticus	**+**	**+**	**+**	**+**
**25**	NRC-118	Nagpur mandarin (*Citrus reticulata)*	Nagpur, Maharashtra	*Ca*. L. asiaticus	**+**	**+**	**+**	**+**
**26**	An-1	Sweet orange (*Citrus sinensis*)	Allahabad, Utter Pradesh	*Ca*. L. asiaticus	**-**	**-**	**-**	**-**
**27**	An-6	Sweet orange (*Citrus sinensis*)	Allahabad, Utter Pradesh	*Ca*. L. asiaticus	**-**	**-**	**-**	**-**
**28**	AP-3	Sour orange (*Citrus aurantium*)	Tirupati, Andhra Pradesh	*Ca*. L. asiaticus	**-**	**-**	**-**	**-**
**29**	AP-4	Pummelo (*Citrus grandis)*	Tirupati, Andhra Pradesh	*Ca*. L. asiaticus	**-**	**-**	**-**	**-**
**30**	Ap-5	Pummelo (*Citrus grandis)*	Tirupati, Andhra Pradesh	*Ca*. L. asiaticus	**-**	**-**	**-**	**-**
**31**	Sm-N-15	Sikkim mandarin (*Citrus reticulata*)	North, Sikkim	*Ca*. L. asiaticus	**-**	**-**	**-**	**-**
**32**	Sm-W-16	Sikkim mandarin (*Citrus reticulata*)	West, Sikkim	*Ca*. L. asiaticus	**-**	**-**	**-**	**-**
**33**	Sm-S-17	Sikkim mandarin (*Citrus reticulata*)	South, Sikkim	*Ca*. L. asiaticus	**-**	**-**	**-**	**-**
**34**	Sm-S-18	Sweet orange (*Citrus sinensis*)	South, Sikkim	*Ca*. L. asiaticus	**-**	**-**	**-**	**-**
**35**	Mmnd-1	Mandarin (*Citrus reticulata*)	Tuivamit, Mizoram	*Ca*. L. asiaticus	**-**	**-**	**-**	**-**
**36**	KP-1	Acid lime (*Citrus aurantifolia*)	Government Nursery, Radhanagari, Kolhapur, Maharashtra	*Ca*. L. asiaticus	**-**	**-**	**-**	**-**
**37**	KP-2	Acid lime (*Citrus aurantifolia*)	Government Nursery, Ajara, Kolhapur, Maharashtra	*Ca*. L. asiaticus	**-**	**-**	**-**	**-**
**38**	KP-3	Acid lime (*Citrus aurantifolia*)	Government Nursery, Chandgad, Kolhapur, Maharashtra	*Ca*. L. asiaticus	**-**	**-**	**-**	**-**
**39**	KP-4	Acid lime (*Citrus aurantifolia*)	Government Nursery, Jaysingpur, Kolhapur, Maharashtra	*Ca*. L. asiaticus	**+**	**+**	**+**	**+**
**40**	KP-5	Acid lime (*Citrus aurantifolia*)	Agriculture College, Kolhapur, Maharashtra	*Ca*. L. asiaticus	**-**	**-**	**-**	**-**
**41**	KP-6	Acid lime (*Citrus aurantifolia*)	Bharat Nursery, Varnul, Kolhapur, Maharashtra	*Ca*. L. asiaticus	**+**	**+**	**+**	**+**
**42**	SGL-1	Acid lime (*Citrus aurantifolia*)	Government NurseryKupwad, Sangli, Maharashtra	*Ca*. L. asiaticus	**-**	**-**	**-**	**-**
**43**	SGL-2	Acid lime (*Citrus aurantifolia*)	Agriculture research center, KasbeDigraj, Sangli, Maharashtra	*Ca*. L. asiaticus	**-**	**-**	**-**	**-**
**44**	ST-1	Sweet orange (*Citrus sinensis*)	Nagpur, Maharashtra	*Ca*. L. asiaticus	**-**	**-**	**-**	**-**
**45**	ST-3	Sweet orange (*Citrus sinensis*)	Nagpur, Maharashtra	*Ca*. L. asiaticus	**-**	**-**	**-**	**-**
**46**	ST-12	Sweet orange (*Citrus sinensis*)	Nagpur, Maharashtra	*Ca*. L. asiaticus	**-**	**-**	**-**	**-**
**47**	ST-16	Sweet orange (*Citrus sinensis*)	Nagpur, Maharashtra	*Ca*. L. asiaticus	**+**	**+**	**+**	**+**
**48**	ST-17	Sweet orange (*Citrus sinensis*)	Nagpur, Maharashtra	*Ca*. L. asiaticus	**-**	**-**	**-**	**-**
**49**	ST-22	Sweet orange (*Citrus sinensis*)	Nagpur, Maharashtra	*Ca*. L. asiaticus	**-**	**-**	**-**	**-**
**50**	HT-2	Sweet orange (*Citrus sinensis*)	Nagpur, Maharashtra	*Ca*. L. asiaticus	**-**	**-**	**-**	**-**
**51**	HT-9	Sweet orange (*Citrus sinensis*)	Nagpur, Maharashtra	*Ca*. L. asiaticus	**-**	**-**	**-**	**-**
**52**	HT-18	Sweet orange (*Citrus sinensis*)	Nagpur, Maharashtra	*Ca*. L. asiaticus	**-**	**-**	**-**	**-**
**53**	HT-23	Sweet orange (*Citrus sinensis*)	Nagpur, Maharashtra	*Ca*. L. asiaticus	**-**	**-**	**-**	**-**
**54**	HT-24	Sweet orange (*Citrus sinensis*)	Nagpur, Maharashtra	*Ca*. L. asiaticus	**-**	**-**	**-**	**-**
**55**	HT-29	Sweet orange (*Citrus sinensis*)	Nagpur, Maharashtra	*Ca*. L. asiaticus	**-**	**-**	**-**	**-**
**56**	HT-31	Sweet orange (*Citrus sinensis*)	Nagpur, Maharashtra	*Ca*. L. asiaticus	**-**	**-**	**-**	**-**
**57**	HT-35	Sweet orange (*Citrus sinensis*)	Nagpur, Maharashtra	*Ca*. L. asiaticus	**-**	**-**	**-**	**-**
**58**	HT-63	Sweet orange (*Citrus sinensis*)	Nagpur, Maharashtra	*Ca*. L. asiaticus	**-**	**-**	**-**	**-**
**59**	HT-72	Sweet orange (*Citrus sinensis*)	Nagpur, Maharashtra	*Ca*. L. asiaticus	**-**	**-**	**-**	**-**
**60**	NY-1	Sweet orange (*Citrus sinensis*)	Nagpur, Maharashtra	*Ca*. L. asiaticus	**-**	**-**	**-**	**-**
**61**	NY-2	Sweet orange (*Citrus sinensis*)	Nagpur, Maharashtra	*Ca*. L. asiaticus	**-**	**-**	**-**	**-**
**62**	NY-3	Sweet orange (*Citrus sinensis*)	Nagpur, Maharashtra	*Ca*. L. asiaticus	**-**	**-**	**-**	**-**
**63**	NY-4	Sweet orange (*Citrus sinensis*)	Nagpur, Maharashtra	*Ca*. L. asiaticus	**-**	**-**	**-**	**-**
**64**	NY-6	Sweet orange (*Citrus sinensis*)	Nagpur, Maharashtra	*Ca*. L. asiaticus	**+**	**+**	**+**	**+**
**65**	NY-7	Sweet orange (*Citrus sinensis*)	Nagpur, Maharashtra	*Ca*. L. asiaticus	**-**	**-**	**-**	**-**
**66**	NY-8	Sweet orange (*Citrus sinensis*)	Nagpur, Maharashtra	*Ca*. L. asiaticus	**+**	**+**	**+**	**+**
**67**	NY-9	Sweet orange (*Citrus sinensis*)	Nagpur, Maharashtra	*Ca*. L. asiaticus	**-**	**-**	**-**	**-**
**68**	NY-10	Sweet orange (*Citrus sinensis*)	Nagpur, Maharashtra	*Ca*. L. asiaticus	**-**	**-**	**-**	**-**
**69**	NY-11	Sweet orange (*Citrus sinensis*)	Nagpur, Maharashtra	*Ca*. L. asiaticus	**-**	**-**	**-**	**-**
**70**	NY-12	Sweet orange (*Citrus sinensis*)	Nagpur, Maharashtra	*Ca*. L. asiaticus	**-**	**-**	**-**	**-**
**71**	NY-13	Sweet orange (*Citrus sinensis*)	Nagpur, Maharashtra	*Ca*. L. asiaticus	**-**	**-**	**-**	**-**
**72**	NY-14	Sweet orange (*Citrus sinensis*)	Nagpur, Maharashtra	*Ca*. L. asiaticus	**+**	**+**	**+**	**+**
**73**	NY-17	Sweet orange (*Citrus sinensis*)	Nagpur, Maharashtra	*Ca*. L. asiaticus	**-**	**-**	**-**	**-**
**74**	NY-24	Sweet orange (*Citrus sinensis*)	Nagpur, Maharashtra	*Ca*. L. asiaticus	**-**	**-**	**-**	**-**
**75**	NY-37	Sweet orange (*Citrus sinensis*)	Nagpur, Maharashtra	*Ca*. L. asiaticus	**-**	**-**	**-**	**-**
**76**	NY-52	Sweet orange (*Citrus sinensis*)	Nagpur, Maharashtra	*Ca*. L. asiaticus	**+**	**+**	**+**	**+**
**77**	NY-54	Sweet orange (*Citrus sinensis*)	Nagpur, Maharashtra	*Ca*. L. asiaticus	**-**	**-**	**-**	**-**
**78**	NY-56	Sweet orange (*Citrus sinensis*)	Nagpur, Maharashtra	*Ca*. L. asiaticus	**+**	**+**	**+**	**+**
**79**	NY-57	Sweet orange (*Citrus sinensis*)	Nagpur, Maharashtra	*Ca*. L. asiaticus	**+**	**+**	**+**	**+**
**80**	NY-60	Sweet orange (*Citrus sinensis*)	Nagpur, Maharashtra	*Ca*. L. asiaticus	**-**	**-**	**-**	**-**
**81**	NY-61	Sweet orange (*Citrus sinensis*)	Nagpur, Maharashtra	*Ca*. L. asiaticus	**+**	**+**	**+**	**+**
**82**	NY-65	Sweet orange (*Citrus sinensis*)	Nagpur, Maharashtra	*Ca*. L. asiaticus	**-**	**-**	**-**	**-**
**83**	NY-66	Sweet orange (*Citrus sinensis*)	Nagpur, Maharashtra	*Ca*. L. asiaticus	**-**	**-**	**-**	**-**
**84**	NY-69	Sweet orange (*Citrus sinensis*)	Nagpur, Maharashtra	*Ca*. L. asiaticus	**-**	**-**	**-**	**-**
**85**	NY-70	Sweet orange (*Citrus sinensis*)	Nagpur, Maharashtra	*Ca*. L. asiaticus	**+**	**+**	**+**	**+**
**86**	NY-104	Sweet orange (*Citrus sinensis*)	Nagpur, Maharashtra	*Ca*. L. asiaticus	**+**	**+**	**+**	**+**
**87**	NY-109	Sweet orange (*Citrus sinensis*)	Nagpur, Maharashtra	*Ca*. L. asiaticus	**-**	**-**	**-**	**-**
**88**	NY-110	Sweet orange (*Citrus sinensis*)	Nagpur, Maharashtra	*Ca*. L. asiaticus	**+**	**+**	**+**	**+**
**89**	CCRI-1	Nagpur mandarin (*Citrus reticulata*)	Nagpur, Maharashtra	Mandarin (Healthy)	**-**	**-**	**-**	**-**
**90**	CCRI-2	Sweet orange (*Citrus sinensis*)	Nagpur, Maharashtra	Mosambi (Healthy)	**-**	**-**	**-**	**-**
**91**	CCRI-3	Acid lime (*Citrus aurantifolia*)	Nagpur, Maharashtra	Acid lime (Healthy)	**-**	**-**	**-**	**-**
**92**	CCRI-4	Pummelo (*Citrus grandis)*	Nagpur, Maharashtra	Pummelo (Healthy)	**-**	**-**	**-**	**-**
**93**	CCRI-5	Grapefruit (*Citrus paradisi*)	Nagpur, Maharashtra	Grapefruit (Healthy)	**-**	**-**	**-**	**-**
**94**	CCRI-6	Assam Lemon (*Citrus limon*)	Nagpur, Maharashtra	Assam Lemon (Healthy)	**-**	**-**	**-**	**-**
**Amplification of non-targeted species (Major citrus viruses and other pathogen)**								
				**Other citrus pathogens**				
**95**	MM-1	Sweet orange (*Citrus sinensis*)	Nagpur, Maharashtra	CMBV	**-**	**-**	**-**	**-**
**96**	MM-2	Sweet orange (*Citrus sinensis*)	Nagpur, Maharashtra	CMBV	**-**	**-**	**-**	**-**
**97**	MM-3	Sweet orange (*Citrus sinensis*)	Nagpur, Maharashtra	CMBV	**-**	**-**	**-**	**-**
**98**	AW-1	Acid lime (*Citrus aurantifolia)*	Nagpur, Maharashtra	CTV	**-**	**-**	**-**	**-**
**99**	AW-2	Acid lime (*Citrus aurantifolia*)	Nagpur, Maharashtra	CTV	**-**	**-**	**-**	**-**
**100**	AW-3	Acid lime (*Citrus aurantifolia)*	Nagpur, Maharashtra	CTV	**-**	**-**	**-**	**-**
**101**	AW-4	Acid lime (*Citrus aurantifolia*)	Nagpur, Maharashtra	CTV	**-**	**-**	**-**	**-**
**102**	AK-1	Kinnow Mandarin (*Citrus reticulata*)	Punjab, Abohar	ICRSV	**-**	**-**	**-**	**-**
**103**	AK-2	Kinnow Mandarin (*Citrus reticulata*)	Punjab, Abohar	ICRSV	**-**	**-**	**-**	**-**
**104**	CK-1	Acid lime (*Citrus aurantifolia*)	Nagpur, Maharashtra	*Xanthomonasaxonopodispv*. *citri*	**-**	**-**	**-**	**-**
**105**	CK-2	Nagpur mandarin (*Citrus reticulata*)	Nagpur, Maharashtra	*Xanthomonasaxonopodispv*. *citri*	**-**	**-**	**-**	**-**
**106**	CK-3	Sweet orange (*Citrus sinensis*)	Nagpur, Maharashtra	*Xanthomonasaxonopodispv*. *citri*	**-**	**-**	**-**	
**107**	Phlp-1	Acid lime (*Citrus aurantifolia*)	Nagpur, Maharashtra	Phytoplasma	**-**	**-**	**-**	**-**
**108**	Phlp-2	Acid lime (*Citrus aurantifolia*)	Nagpur, Maharashtra	Phytoplasma	**-**	**-**	**-**	**-**

Note: All mentioned samples were checked with, HLB-RPA-LFA (Visualization on PCRD nucleic acid detector), HLB-RPA with primer set DKG-F/DKG-R (Visualization using agarose gel), conventional PCR with RPA primers set DKG-F/DKG-R and conventionalPCR with primer set OI1/OI2c.

## Discussion

Huanglongbing has decimated citrus industries worldwide and killed millions of citrus plants globally [8]. In India, the presence of HLB was recorded in 1966 and in the following years reported from all citrus growing states. In India, citrus is mainly cultivated by vegetative propagation. The unavailability of natural resistance to HLB in commercial cultivars and/or chemical control measures makes citrus greening disease (HLB) a major threat to the Indian citrus industry. Use of quick, sensitive, robust and low cost detection techniques is the most viable strategy for early diagnosis that can be used to prevent subsequent transmission of the disease.

Piepenburg et al., (2006) reported recombinase polymerase amplification as the best suited technique for point-of-care diagnostics by little or low cost instrumentation facilities[27]. Therefore, in the present study, an HLB-RPA-LFA assay has been established for speedy visual detection of ‘*Ca*. L. asiaticus’ which requires minimum instrumentation and has the potential to be used as a point of care diagnostic tool. The most important practicality of this technique is the shorter detection time. It requires only 20 to 25 min for DNA amplification and to detect the RPA products by agarose gel electrophoresis requires 60 to 80 min. The specificity of the RPA primers was cross verified by using conventional PCR and sequencing of purified PCR products, which showed the selective amplification of the ‘*Ca*. L. asiaticus’. To obtain better visibility by agarose electrophoresis, after completion of the reaction, the RPA product was purified using chloroform/isoamyl alcohol extraction. This additional step gives clear visibility as compared to unpurified products on the agarose gel. A very low amplification background was observed on agarose gel in positive plant and which was not observed in case of HLB negative plant (Fig 5). Similar reports of low amplification background in RPA technique was also observed in various study [40, 41, 29]and it does not affect the specificity of primers because the same primer pair did not amplify the expected size amplicon and low amplification background as well in DNA extracted from healthy samples. The specificity of RPA primers also investigated by *in silico* analysis *viz*. primer BLAST and *in silico* PCR. It is observed that primers are specific to target pathogen and do not cross react with other major citrus pathogens.

The sensitivity of RPA was shown to be better than conventional PCR however it is less sensitive as compare to TaqMan-qPCR (Figs 9, 10 and S1 Fig). Additionally, PCR reactions were conducted by using the RPA primers and these primers could also be used in conventional PCR. For development of HLB-RPA-LFA the TwistAmpnfo kit and PCRD nucleic acid detector were used. For positive HLB-RPA-LFA, both test and control lines were visible whereas if the assay was negative, only the control line was visible. For higher concentration of pathogen, the test line was visible after 3 min if the pathogen had a higher titer while at lesser concentrations of the pathogen it took up to 5 min. The optimal amplification temperature of the RPA assay ranged between 37°C and 42°C and it was observed that the best result occurred at 38°C. Similarly, others have reported that RPA tolerated temperature fluctuations in the range of 25°C and 42°C[42, 43]without the performance of the reaction being compromised. This would suggest that precise detection can be achieved in the field by a simple battery-powered portable instrument reducing the cost of detection [44].

The HLB-RPA-LFA presented in this study was validated by the conventional PCR, 16S rDNA-based OI1/OI2c and rplA-rpl J region specific A2/J5 primers (Figs 2 and 3), TaqMan-qPCR and AmplifyRP Acceler8 Las detection kit. This commercially available kit is expensive and not affordable for the farmers. Hence, there is need for the development of an affordable indigenous kit for detection of devastating pathogens. In India, citrus crops are affected by numerous virus and virus-like pathogens including *Citrus tristeza virus* (CTV), *Citrus yellow mosaic badna virus*(CMBV), *Indian citrus ringspot virus* (ICRSV), *Citrus exocortis viroid*[8]. The specificity of HLB-RPA-LFA was confirmed by testing eighty-eight samples of different citrus cultivars for HLB and compared these results with conventional PCR, PCR with RPA primers and HLB-RPA (Table 3) based on DNA as a template. The cross-reactivity was not observed in HLB-RPA-LFA with other pathogens infecting citrus and this confirmed the specificity of the newly-designed RPA primers and probes.

Another isothermal assay, HLB-Loop mediated isothermal amplification is also available for rapid diagnosis of disease but require comparatively more time. RPA is simple and can be completed within 20–30 min. However, HLB-RPA-LFA would be the preferred nucleic acid based-diagnostic tool to use where expensive molecular biology instruments such as Real time PCR machines, PCR machines, centrifuges, electrophoresis and gel documentation units are not required. The cost of required consumables for HLB-RPA-LFA technique can be reduced by developing an indigenous low cost nucleic acid detector (unpublished data). The developed HLB-RPA-LFA assay is a simple, rapid, user-friendly and sensitive method for detection of ‘*Ca*. L. asiaticus’ and has great potential in future to serve as a better diagnostic tool for farmers, nurserymen, disease surveyors and mobile plant pathology laboratories. To the best of our knowledge, this is the first report of successful development HLB-RPA-LFA for the detection ‘*Ca*. L. asiaticus’.

## Supporting information

S1 File TaqMan-qPCR assay. Designing of primers, probe and assay conditions.
(DOCX)

S1 Fig Detection limit of TaqMan-qPCR. Amplification plot generated using 10-foldserial dilution of total genomic DNA (known concentration) of Mosambi plants infected with *C*Lasto cross verify sensitivity of TaqMan-qPCR with RFA-LFA using HLBas-F/R-HLBp primer probe pair, Line-a = 10ng, Line-b = 1 ng, Line-c = 0.1 ng, Line-d = 0.01 ng, Line-e = 1 pg and Line-f = 100 fg template DNA.
(TIF)

S1 Table Total number of *Candidatus* species and accession number of sequences used for *in silico* PCR analysis. (DOCX)

## References

[1] Bove JM. Hunglongbing: a destructive, newly emerging, century-old disease of citrus. J. Plant Pathol. 2006; 88:7–37.

[2] Ghosh DK, Bhose S, Motghare M, Warghane A, Mukherjee K, Ghosh DK Sr, Sharma AK, Ladaniya MS, Gowda S. Genetic diversity of the Indian populations of ‘*Candidatus* liberibacter asiaticus’ based on the tandem repeat variability in a genomic locus. Phytopathology. 2015; 105:1043–1049. 10.1094/PHYTO-09-14-0253-R 25760522 10.1094/PHYTO-09-14-0253-R

[3] Wang N, Trivedi P. Citrus Huanglongbing: A newly relevant disease presents unprecedented challenges. Phytopathology. 2013; 103:652–665. 10.1094/PHYTO-12-12-0331-RVW 23441969 10.1094/PHYTO-12-12-0331-RVW

[4] Iftikhar Y, Rauf S, Shahzad U, Muhammad AZ. Huanglongbing: Pathogen detection system for integrated disease management—A review. J. Saudi Soci of Agri Sciences. 2016; 15:1–11.

[5] Da Graca JV, Douhan GW, Halbert SE, Keremane ML, Lee RF, Vidalakis G, Zhao H. Huanglongbing: An overview of a complex pathosystem ravaging the world’s citrus. J. Integr. Plant Biol. 2016; 58:373–387. 10.1111/jipb.12437 26466921 10.1111/jipb.12437

[6] Ghosh DK, Bhose S, Mukherjee K, Baranwal VK. Sequence and evolutionary analysis of ribosomal DNA from Huanglongbing (HLB) isolates of Western India. Phytoparasitica. 2013; 4:295–305.

[7] Warghane A, Misra P, Shukla PK, Ghosh DK. Diversity and characterization of *Citrus tristeza virus* and ‘*Candidatus* Liberibacter asiaticus’ associated with citrus decline in major citrus growing areas of India. Indian Phytopathol. 2017; 70:359–367.

[8] Ahlawat YS. Virus diseases of Citrus and Management. 1st ed Studium Press (India) Pvt Ltd; 2012.

[9] Gottwald TR, Da Graça JV, Bassanezi RB. Citrus Huanglongbing: The pathogen and its impact. Plant Health Progress 2007 Online. Plant Health Progress. 10.1094/PHP-2007-0906-01-RV

[10] Batool A, Iftikhar Y, Mughal SM, Khan MM, Jaskani MJ, Abbas M, and Khan IA. Citrus Greening Disease–A major cause of citrus decline in the world—A Review. Hort Sci. 2007; 34:159–166.

[11] Da Graca JV. Citrus greening disease. Ann. Review Phytopathology. 1991; 29:109–136.

[12] Garnier M, Marti-Gros G, Bove JM. Monoclonal antibodies against the bacterial like organism associated with the citrus greening disease. Ann Inst Pasture Microbiol. 1987; 138:639–650. 10.1016/0769-2609(87)90142-6 3331293

[13] Pagliaccia D, Shil J, Pang Z, Hawara E, Clark K, Thapa SP, De Francesco A, Liu J, Tran T-T, Bodaghi S, Folimonova SY, Ancona V, Mulchandani A, Coaker G, Wang N, Vidalakis G, Ma W.A pathogen secreted protein as a detection marker for citrus huanglongbing. Front. Microbiol. 2017; 8:2041 10.3389/fmicb.2017.02041 29403441 10.3389/fmicb.2017.02041 PMC5776943

[14] Jagoueix S, Bove JM, Garnier M. PCR detection of the two ‘*Candidatus*liberobacterspecies’ associated with greening disease of citrus. Mol. Cell. Probes. 1996; 10:43–50. 10.1006/mcpr.1996.0006 8684375 10.1006/mcpr.1996.0006

[15] Li W, Hartung JS, Levy L. Quantitative real-time PCR for detection and identification of ‘*Candidatus* Liberibacter’ species associated with citrus Huanglongbing. J. Microbiol. Meth. 2006; 66:104–115. 10.1016/j.mimet.2005.10.018 16414133

[16] Wang Z, Yin Y, Hu H, Yuan Q, Peng G, Xia Y. Development and application of molecular‐based diagnosis for ‘*Candidatus* Liberibacter asiaticus’, the causal pathogen of citrus huanglongbing. Plant Pathology. 2006; 55:630–8.

[17] Li W, Levy L, Hartung JS. Quantitative distribution of ‘*Candidatus* Liberibacter asiaticus’ in citrus plants with citrus huanglongbing. Phytopathology. 2009; 99:139–44. 10.1094/PHYTO-99-2-0139 19159305 10.1094/PHYTO-99-2-0139

[18] Morgan JK, Zhou L, Li W, Shatters RG, Keremane M, Duan YP. Improved real-time PCR detection of '*Candidatus* Liberibacter asiaticus’ from citrus and psyllid hosts by targeting the intragenic tandem-repeats of its prophage genes. Mol Cell Probes. 2012; 26:90–8. 10.1016/j.mcp.2011.12.001 22245034 10.1016/j.mcp.2011.12.001

[19] Hu H, Davis MJ, Brlansky RH. Quantification of live ‘*Candidatus* Liberibacter asiaticus’ populations using real-time PCR and propidium monoazide. Plant Dis.2013; 97:1158–1167. 10.1094/PDIS-09-12-0880-RE 30722419

[20] Villechanoux S, Garnier M, Renaudin J, Bove JM. Detection of several strains of the bacterium-like organism of citrus greening disease by DNA probes. Curr. Microbio. 1992; 24: 89–95.

[21] Okuda M, Matsumoto M, Tanaka Y, Subandiyah S, Iwanami T. Characterization of the tufB-secE-nusG-rplKAJL-rpoB gene cluster of the citrus greening organism and detection by loop-mediated isothermal amplification. Plant Dis. 2005; 89:705–711. 10.1094/PD-89-0705 30791239

[22] Ghosh DK, Bhose S, Warghane A, Motghare M, Sharma AK, Dhar AK, Gowda S. Loop-mediated isothermal amplification LAMP based method for rapid and sensitive detection of ‘*Candidatus* Liberibacter asiaticus’ in citrus and the psyllid vector, *Diaphorinacitri* Kuwayama. J. Plant Biochem. Biotech. 2016; 25:219–223.

[23] Hosam Z, Mahmoud ES. Recombinase polymerase amplification as a promising tool in Hepatitis C virus diagnosis. World. J Hepatol. 2014; 6:916–922. 10.4254/wjh.v6.i12.916 25544878 10.4254/wjh.v6.i12.916 PMC4269910

[24] Boyle D, McNerney R, Teng Low H, Leader B, Pérez-Osorio A. Rapid detection of mycobacterium tuberculosis by recombinase polymerase amplification. PLoS ONE. 2014; 9(8): e1030919 10.1371/journal.pone.0103091 eCollection 2014. 25118698 10.1371/journal.pone.0103091 PMC4138011

[25] Abukhalid N, Pastey MK. Nucleic acid amplification using recombinase polymerase: enzymatic approach. J. Med. Microb. Diagn. 2017; 6:1–3. 10.4172/2161-0703.1000250

[26] Hou P, Zhao G, Wang H, He C, Huan Y, He H. Development of a recombinase polymerase amplification combined with lateral-flow dipstick assay for detection of bovine ephemeral fever virus. Mol. Cell. Probe. 2018; 38:31–37. 10.1016/j.mcp.2017.12.003 PMC7126596 29288049

[27] Silva G, Oyekanmi J, Nkere CK, Bömer M, Kumar PL, Seal SE. Rapid detection of potyviruses from crude plant extracts. Anal. Biochem. 2018; 546:17–22. 10.1016/j.ab.2018.01.019 29378167 10.1016/j.ab.2018.01.019 PMC5873530

[28] Timothy DM, Frank NM, Michael DC, Timothy DM, Frank NM, Michael DC. Development of rapid isothermal amplification assays for detection of phytophthora spp. in plant tissue. Phytopathology. 2015; 105:265–278. 10.1094/PHYTO-05-14-0134-R 25208239 10.1094/PHYTO-05-14-0134-R

[29] Kumar PV, Sharma SK, Rishi N, Ghosh DK, Baranwal VK. An isothermal based recombinase polymerase amplification assay for rapid, sensitive and robust indexing of *Citrus yellow mosaic virus*. Acta Virol. 2018; 62:104–108. 10.4149/av_2018_113 29521109 10.4149/av_2018_113

[30] Londoño MA, Harmon CL, Polston JE. Evaluation of recombinase polymerase amplification for detection of begomoviruses by plant diagnostic clinics. Virol. J. 2016; 13:48 10.1186/s12985-016-0504-8 eCollection 2014. 27000806 10.1186/s12985-016-0504-8 PMC4802622

[31] Qian W, Lu Y, Meng Y, Ye Z, Wang L, Wang R, Zheng Q, Wu H, Wu J. Field detection of citrus huanglongbing associated with ‘Candidatus liberibacter asiaticus’ by recombinesepolymerase amplification within 15 min. Journal ofAgricultural and Food Chemistry 2018; 66:5473–5480. 29781618 10.1021/acs.jafc.8b01015

[32] Piepenburg O, Williams CH, Stemple DL, Armes NA. DNA detection using recombination proteins. PLoS Biol. 2006; 4 (7): e204 10.1371/journal.pbio.0040204 16756388 PMC1475771

[33] Ye J, Coulouris G, Zaretskaya I, Cutcutache I, Rozen S, and Madden T. Primer-BLAST: A tool to design target-specific primers for polymerase chain reaction. BMC Bioinformatics 2012; 13:134 10.1186/1471-2105-13-134 22708584 10.1186/1471-2105-13-134 PMC3412702

[34] Bikandi J, San MR, Rementeria A, Garaizar J. *In silico* analysis of complete bacterial genomes: PCR, AFLP-PCR, and endonuclease restriction. Bioinformatics 2004; 20:798–9. 10.1093/bioinformatics/btg491 14752001 10.1093/bioinformatics/btg491

[35] Bhose S, Misra P, Ramteke PW, Ghosh D. Sequence analysis of ribosomal protein gene of '*Candidatus* Liberibacter asiaticus’ infecting major citrus cultivars in western Maharashtra of India. Indian Phytopath. 2015; 68:334–341.

[36] Hocquellet A, Toorawa P, Bove JM, Garnier M. Detection and identification of the two *Candidatus* Liberobacter species associated with citrus huanglongbing by PCR amplification of ribosomal protein genes of the beta operon. Mol. Cell. Probes 1999; 13: 373–379. 10.1006/mcpr.1999.0263 10508559 10.1006/mcpr.1999.0263

[37] Li W, Hartung JS, Levy L. Quantitative real-time PCR for detection and identification of *Candidatus* Liberibacter species associated with citrus huanglongbing. J. Microbiol. Methods. 2006; 66:104–115. 10.1016/j.mimet.2005.10.018 16414133 10.1016/j.mimet.2005.10.018

[38] Li W, Li D, Twieg E, Hartung JS, Levy L. Optimized quantification of unculturable *Candidatus* Liberibacter species in host plants using real-time PCR. Plant Dis. 2008;92:854–861. 10.1094/PDIS-92-6-0854 30769724

[39] Ghosh DK, Kokane S, Kumar P, Ozcan A, Warghane A, Motghare M, Santra S, Sharma AK. Antimicrobial nano-zinc oxide-2S albumin protein formulation significantly inhibits growth of ‘*Candidatus* Liberibacter asiaticus’ in planta. PLoS ONE 2018; 13(10): e0204702 10.1371/journal.pone.0204702 30304000 PMC6179220

[40] Rosser A, Rollinson D, Forrest M, Webste BL. Isothermal Recombinase Polymerase amplification (RPA) of *Schistosoma haematobium* DNA and oligochromatographic lateral flow detection. Parasites &Vectors 2015; 8:446. 26338510 10.1186/s13071-015-1055-3 PMC4559068

[41] Kapoor R, Srivastava N, Kumar S, Saritha RK, Sharma SK, Jain RK, Baranwal VK. Development of a recombinase polymerase amplification assay for the diagnosis of banana bunchy top virus in different banana cultivars. Arch Virol. 2017; 162:2791–2796. 10.1007/s00705-017-3399-9 28500444 10.1007/s00705-017-3399-9

[42] Chandu D, Paul S, Parker M, Dudin Y, King-Sitzes J, Perez T, Mittanck D, Shah M, Glenn KC, Piepenburg O. Development of a rapid point-of-use DNA test for the screening of genuity roundup ready 2 yield soybean in seed samples. BioMed Res International. 2016; 1–12. 10.1155/2016/3145921. PMC4899603 27314015

[43] Daher RK, Stewart G, Boissinot M, Bergeron MG. Recombinase polymerase amplification for diagnostic applications. Clin. Chem. 2016; 62:947–958. 10.1373/clinchem.2015.245829 27160000 10.1373/clinchem.2015.245829 PMC7108464

[44] Lillis L, Lehman D, Singhal MC, Cantera J. Singleton J, Labarre P, Toyama A, Piepenburg O, Parker M, Wood R, Overbaugh J, Boyle DS. Non-instrumented incubation of a recombinase polymerase amplification assay for the rapid and sensitive detection of proviral HIV-1 DNA. PLoS ONE. 2014; 9(9): e108189 10.1371/journal.pone.0108189 25264766 PMC4180440

