# Supplementary material for: Development of a recombinase polymerase based isothermal amplification combined with lateral flow assay (HLB-RPA-LFA) for rapid detection of "Candidatus Liberibacter asiaticus"
Source: PLoS One. 2018 Dec 12;13(12):e0208530. doi: 10.1371/journal.pone.0208530 (PMC6291142; doi:10.1371/journal.pone.0208530)
Supplement: S1 File — Designing of primers, probe and assay conditions. (DOCX) [file pone.0208530.s001.docx]

**Supporting Information**

**TaqMan-qPCR assay**

**Designing of probe and primers**

TaqMan-qPCR was performed to validate the sensitivity of RPA-LFA technique using *C*Las specific primer pair, a forward primer HLBas-F (5’-TCGAGCGCGTATGCAATACG -3’), reverse primer HLBas-R (5’-GCGTTATCCCGTAGAAAAAGGTAG-3’) and probe, HLB-p (5’-56-FAM/AGACGGGTGAGTAACGCG/3BHQ_1-3’) based on sequences of 16S rDNA with an expected amplicons length of 76 bp (GenBank accession number L22532) [37,38,39]. The 5’ terminal end of HLB-probe was labeled with 6-carboxy-fluorescein (FAM) reporter dye and 3’ terminal end nucleotide with Black Hole Quencher (BHQ)-1dye.

**TaqMan-real time PCR assay conditions**

The assay was performed using a StepOne Real Time PCR System (Applied Biosystems) in a total of 10 µl reaction volume consisting of 300 nM each forward and reverse primers, 200 nM probe with 1x TaqMan Universal Master Mix II (Applied Biosystems). The protocol was 95°C for 10 min (initial denaturation), followed by 40 cycles at 95°C for 15 s, annealing and primer extension simultaneously for 1 min at 60°C. The data were analyzed using StepOne Software v2.1 and showing Ct value, 23.29, 18.78, 22.23, 22.76, 21.50 and 20.62 for collected HLB positive sample C1 to C6 respectively whereas undetermined Ct observed for non template control (Fig 12). It is observed that TaqMan-real time PCR has a limit of detection less than 100 fg and up to 10 fg which is very high as compared to RPA-LFA and conventional PCR.
