# Supplementary figures and images for: Development of a recombinase polymerase based isothermal amplification combined with lateral flow assay (HLB-RPA-LFA) for rapid detection of "Candidatus Liberibacter asiaticus"
Source: PLoS One. 2018 Dec 12;13(12):e0208530. doi: 10.1371/journal.pone.0208530 (PMC6291142; doi:10.1371/journal.pone.0208530)

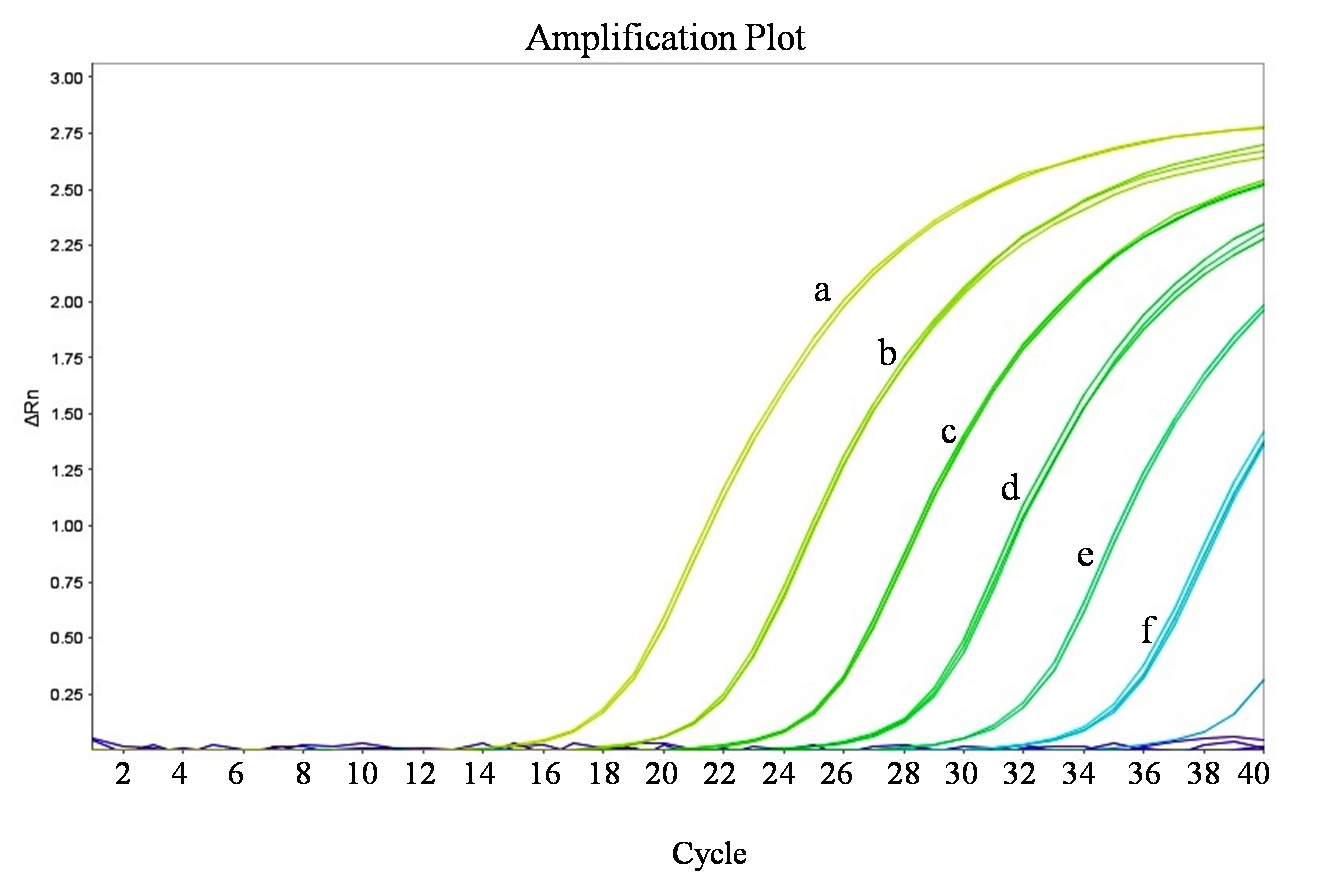

Supplement: S1 Fig — Amplification plot generated using 10-foldserial dilution of total genomic DNA (known concentration) of Mosambi plants infected with CLasto cross verify sensitivity of TaqMan-qPCR with RFA-LFA using HLBas-F/R-HLBp primer probe pair, Line-a = 10ng, Line-b = 1 ng, Line-c = 0.1 ng, Line-d = 0.01 ng, Line-e = 1 pg and Line-f = 100 fg template DNA. (TIF) [file pone.0208530.s002.tif]
