# Supplementary material for: Development of a recombinase polymerase based isothermal amplification combined with lateral flow assay (HLB-RPA-LFA) for rapid detection of "Candidatus Liberibacter asiaticus"
Source: PLoS One. 2018 Dec 12;13(12):e0208530. doi: 10.1371/journal.pone.0208530 (PMC6291142; doi:10.1371/journal.pone.0208530)
Supplement: S1 Table — (DOCX) [file pone.0208530.s003.docx]

**S1 Table. Total number of *Candidatus* species and accession number of sequences used for *in silico* PCR analysis.**

| **S. No** | ***Candidatus* Species** | **Accession number** |
| --- | --- | --- |
| 1 | *Candidatus* Zinderia insecticola CARI | CP002161 |
| 2 | *Candidatus* Accumulibacter phosphatis clade IIA str. UW-1 | NC_013194 |
| 3 | *Candidatus* Amoebophilus asiaticus5a2 | CP001102 |
| 4 | *Candidatus* Arthromitus sp. SFB-mouse-Japan | AP012202 |
| 5 | *Candidatus* Arthromitus sp. SFB-rat-Yit | AP012210 |
| 6 | *Candidatus* Arthromitus sp. SFB-mouse-Yit | AP012209 |
| 7 | *Candidatus* Azobacteriosides pseudotrichonymphae genomovar. CFP2 | NC_011565 |
| 8 | *Candidatus* Blochmannia chromaiodes str.640 | CP003903 |
| 9 | *Candidatus* Blochmannia vafer str. BVAF | NC_014909 |
| 10 | *Candidatus* Caldiarchaeum subterraneum from subsurface mine microbial mat | BA000048 |
| 11 | *Candidatus* Carsonella ruddii CE isolate Thao2000 | CP003541 |
| 12 | *Candidatus* Carsonella ruddii DC | CP003467 |
| 13 | *Candidatus* Carsonella ruddii HC isolate Thao2000 | CP003543 |
| 14 | *Candidatus* Carsonella ruddii HT isolate Thao2000 | CP003544 |
| 15 | *Candidatus* Carsonella ruddii CS isolate Thao2000 | CP003542 |
| 16 | *Candidatus* Carsonella ruddii PC isolate NHV | CP003545 |
| 17 | *Candidatus* Carsonella ruddii PV | AP009180 |
| 18 | *Candidatus* Chloracidobacterium thermophilum B | GCF_000226295.1 |
| 19 | *Candidatus* Cloacamonas acidaminovorans str. Evry provisional genome sequence from WWE1 candidate division | NC_020449 |
| 20 | *Candidatus* Desulfococcus oleovorans Hxd3 | CP000859 |
| 21 | *Candidatus* Desulforudis audaxviator MP104C | NC_010424 |
| 22 | *Candidatus* Hamiltonella defense 5AT(Acyrthosiphon pisum) | CP001277 |
| 23 | *Candidatus* Hodgkinia cicadicola Dsem | CP001226 |
| 24 | *Candidatus* Kinetoplastibacterium blastocrithidii (ex Strigomonas culicis) | CP003733 |
| 25 | *Candidatus* Kinetoplastibacterium crithidi (ex Angomonas deanei ATCC 30255) | CP003978 |
| 26 | *Candidatus* Kinetoplastibacterium blastocrithidii TCC012E | CP003807 |
| 27 | *Candidatus* Kinetoplastibacterium desouzaii TCC079E | CP003803 |
| 28 | *Candidatus* Kinetoplastibacterium galatii TCC219 | CP003806 |
| 29 | *Candidatus* Kinetoplastibacterium oncopeltii TCC079E | - |
| 30 | *Candidatus* Korarchaeum cryptofilum OPF8 | CP000968 |
| 31 | *Candidatus* koribacter versatilis Ellin345 | NC_008009 |
| 32 | *Candidatus* Liberibacter americanus str. Sao Paulo | P006604 |
| 33 | ***Candidatus* Liberibacter asiaticus str. Gxpsy** | CP004005 |
| 34 | ***Candidatus* Liberibacter asiaticus str. Psy62** | NC_012985 |
| 35 | *Candidatus* Liberibacter solanacearum Clso-ZC1 | NC_014774 |
| 36 | *Candidatus* Mathanomethylophilus alvus Mx1201 | CP004049 |
| 37 | *Candidatus* Methanosphaerula palustris E1-9c | NC_011832 |
| 38 | *Candidatus* Methylomirabilis oxyfera | NSJN01000001 |
| 39 | *Candidatus* Midichloria mitochondrii iricVA | NC_015722 |
| 40 | *Candidatus* Moranella endobia PCIT | NC_015735 |
| 41 | *Candidatus* Moranella endobia PCVAL | NC_021057 |
| 42 | *Candidatus* Mycoplasma haemolame str. Purdue | CP003731 |
| 43 | *Candidatus* Mycoplasma haemominutum Birmingham 1 noncontiguous finished genome | NC_021007 |
| 44 | *Candidatus* Nausia deltocephalinicola str. NAS-ALF | CP006059 |
| 45 | *Candidatus* Nitrosopumilus koreensis AR1 | CP003842 |
| 46 | *Candidatus* Nitrosopumilus sp. AR2 | CP003843 |
| 47 | *Candidatus* Nitrososphaera gargensis Ga9.2 | CP003843 |
| 48 | *Candidatus* Nitrosopira defluvii | FP929003 |
| 49 | *Candidatus* Pelagibacter sp. IMCC9063 | CP002511 |
| 50 | *Candidatus* Phytoplasma australiense | DQ318777 |
| 51 | *Candidatus* Phytoplasma mali | NC_011047 |
| 52 | *Candidatus* Portiera aleyrodidarum BT-B | NC_018507. |
| 53 | *Candidatus* Portiera aleyrodidarum BT-B-HRs | HG788363 |
| 54 | *Candidatus* Portiera aleyrodidarum BT-Q-AWR | *-* |
| 55 | *Candidatus* Portiera aleyrodidarum BT-QVLC | CP003835 |
| 56 | *Candidatus* Portiera aleyrodidarum TV | CP004358 |
| 57 | *Candidatus* Rickettsia amblyommii str. GAT-30V | CP003334 |
| 58 | *Candidatus* Riesia pediculicola USDA | NC_014109 |
| 59 | *Candidatus* Ruthia magnifica str. Cm (Calyptogena magnifica) | CP000488 |
| 60 | *Candidatus* Saccharibacteria bacterium RAAC3_TM7_1 | CP006915 |
| 61 | *Candidatus* Saccharimonas aalborgensis | CP005957 |
| 62 | *Candidatus* Sulcia muelleri CARI | CP002163 |
| 63 | *Candidatus* Sulcia muelleri DMIN | CP001981 |
| 64 | *Candidatus* Sulcia muelleri GWSS | HG787070 |
| 65 | *Candidatus* Sulcia muelleri SMDSEM | CP001605 |
| 66 | *Candidatus* Sulcia muelleri str. Sulcia-ALF | CP006060 |
| 67 | *Candidatus* Tremblaya phenacola PAVE | CP003982 |
| 68 | *Candidatus* Tremblaya princeps PCIT | CP002244 |
| 69 | *Candidatus* Tremblaya princeps PCVAL | CP002918 |
| 70 | *Candidatus* Uzinura diaspidicola str. ASNER | CP003263 |
| 71 | *Candidatus* Vesicomyosocius okutanii HA | NC_009465 |
| 72 | *Candidatus* Kinetoplastibacterium crithidii TCC036E | JF756595 |
